# Age and sex effects in physical fitness components of 108,295 third graders including 515 primary schools and 9 cohorts

**DOI:** 10.1038/s41598-021-97000-4

**Published:** 2021-09-02

**Authors:** Thea Fühner, Urs Granacher, Kathleen Golle, Reinhold Kliegl

**Affiliations:** Division of Training and Movement Sciences, Research Focus Cognition Sciences, University of Potsdam, Am Neuen Palais 10, Building 12, 14469 Potsdam, Germany

**Keywords:** Human behaviour, Statistics

## Abstract

Children’s physical fitness development and related moderating effects of age and sex are well documented, especially boys’ and girls’ divergence during puberty. The situation might be different during prepuberty. As girls mature approximately two years earlier than boys, we tested a possible convergence of performance with five tests representing four components of physical fitness in a large sample of 108,295 eight-year old third-graders. Within this single prepubertal year of life and irrespective of the test, performance increased linearly with chronological age, and boys outperformed girls to a larger extent in tests requiring muscle mass for successful performance. Tests differed in the magnitude of age effects (gains), but there was no evidence for an interaction between age and sex. Moreover, “physical fitness” of schools correlated at r = 0.48 with their age effect which might imply that "fit schools” promote larger gains; expected secular trends from 2011 to 2019 were replicated.

## Introduction

Children’s development of physical fitness as well as the effects of moderating variables such as age and sex are well documented^1–7^, especially boys’ and girls’ divergence during puberty starting late in the twelfth and tenth year of life, respectively^8^. The situation might be different during prepuberty. Girls mature visibly about two years earlier than boys, but sex hormones rise already much starting at eight years of age^9–11^. If the early rise of sex hormones relates to body composition, the question arises whether there is evidence for faster development of girls than boys in this year, leading to a convergence of performance in physical fitness during the transition from pre-puberty to puberty?

**Figure 1:**
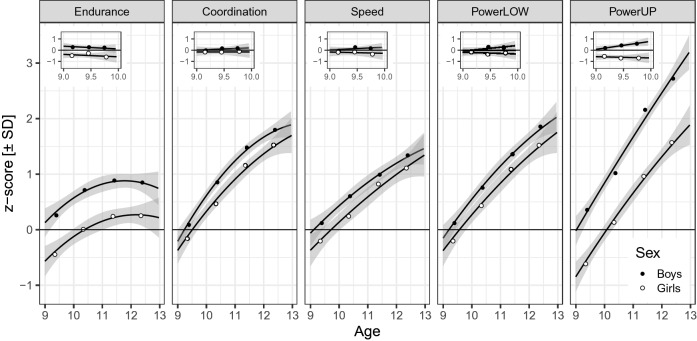
Physical fitness curves of a longitudinal sample of 240 German boys (closed circles) and girls (open circles) followed from age 9 to 12 years for endurance = cardiorespiratory endurance (i.e., 9 min run test), coordination (i.e., running in a star like pattern), speed (i.e., 50-m linear sprint test), powerLOW = power of lower limbs (i.e., triple hop test), and powerUP = power of upper limbs (i.e., ball push test). The insets show for each test score the regression on age for the first assessment when children were between 9.00 and 9.99 years old. Also shown are the means for groups of boys and girls binned into three age groups (i.e., 9.00–9.33; 9.34–9.66; 9.67–9.99). Error bands are 95% CIs. Data are from Golle et al.^6^.

A longitudinal study on the development of different fitness components highlights sex-specific performance trajectories in youth aged 9–12 years^6^. This study was a precursor project of the present cross-sectional study and tested four components of physical fitness in four annual assessments including 240 children^6^. The main panels in Fig. 1 illustrate sex- and age-differential development for cardiorespiratory endurance, coordination, speed, and power (assessed separately for lower [powerLOW] and upper [powerUP] limbs). The five tests used for the assessment are described in the figure caption.

Speed, powerLOW, and powerUP follow a mostly linear trajectory, whereas cardiorespiratory endurance and coordination are characterized by a curvilinear development^6^. These age-related differences can mostly be attributed to growth (increasing body mass and body height) and maturation during childhood and adolescence^1^. For instance, while increased body mass may have a positive impact on ball push test performance, it may negatively influence performance in tests such as the 6 or 9 min run tests which afford the continuous acceleration of the body^12^. Furthermore, the fact that boys significantly outperform girls in these tests^6^ is due to differences in muscle mass favouring boys; there is evidence that prepuberal boys have on average 3.7% larger muscle mass compared with girls^1,13^. In line with this argument, Golle et al.^6^ observed larger sex differences in fitness tests demanding muscle mass (e.g., powerUP) compared with tests of motor coordination (e.g., running in a starlike pattern).

The longitudinal study of Golle et al.^6^ and other cross-sectional studies^4,5,7,14,15^ showed performance increases for all components of physical fitness over childhood and adolescents. However, it may come as a surprise that in the longitudinal study of Golle et al.^6^ none of the *cross-sectional* age differences and none of the interactions with sex were significant *within* the respective assessment years—neither when aggregated over tests nor when tested for individual tests; the insets in Fig. 1 show these non-significant trends and the means for three age groups for the first assessment of tests when children were between 9.00 and 9.99 years (see Supplement B for details of a reanalysis). However, the absence of evidence for cross-sectional age differences and their interactions with sex within a single year of life at a prepubertal development stage must not be taken as evidence for their absence^16^, because statistical power may not have been sufficiently large.

Our study included a very large representative sample of 108,295 eight-year old third graders. This large sample allowed us to zoom into a cross-sectional *short-term ontogenetic* window. In this single year of life, we expected to detect age differences that were not significant in the insets of Fig. 1. Indeed, we expected that the component differences in developmental gains (i.e., the differences in slopes) will be in general agreement with those in the main panel of Fig. 1 because gains should be larger (favouring older children) in tests that do not demand continuous acceleration of the body as with the ball push test vs the 6 min run test^12^. Similarly, as in Fig. 1 (both in the main panel and in the insets) the test-related sex effects (i.e., the differences between the lines) will be larger, the more muscle mass is required to perform a physical fitness test^1,13^ (favouring boys) and less for tests involving motor coordination^17^. In other words, in general, we expected to anticipate the *long-term ontogenetic longitudinal trends* across four years with *short-term ontogenetic cross-sectional trends* within a single year for younger children in the third grade*.*

There is one exception to this expectation of agreement between *short-term* and *long-term ontogenetic* trends: The only significant interaction between age and sex was reported for powerUP (see right panel in Fig. 1) indicating divergence between boys and girls. If physical fitness components carry an early prepubertal signal in eight-year olds, then we should observe convergence of scores because girls will benefit earlier than boys from the rise of sex hormones^9,11^.

Cross-sectional analysis has been criticized for good reasons and in general preference is given to longitudinal analysis, mostly because only the latter delivers information about intraindividual growth^6^. However, we propose that, when the focus is on *short-term ontogenesis,* that is on changes *within*, not *between* years of life, a cross-sectional design is probably the only option to determine development-related gains because this design circumvents practice effects, a necessary consequence of repeated testing of physical fitness within a year. Another problem of cross-sectional designs are cohort effects (i.e., age-correlated cultural change). However, cohort effects are certainly negligible for children who attend the same grade but differ in age only within the same year of life. Thus, with the exception of loss of information about interindividual differences in intraindividual change, a cross-sectional design is more suitable to determine how within-year developmental profiles differ between *health*-*related* (e.g., cardiorespiratory endurance)^18^ and *skill-related* components of physical fitness (e.g., power, speed, coordination)^18^.

Just as there are individual differences in physical fitness between children, there are also differences between the over 500 schools in how much they implement programs that facilitate gains in the development of physical fitness^19^. Explanatory hypotheses about these differences are beyond the scope of this article, but our use of a linear mixed model for statistical inference affords exploratory tests for their presence and adjusts test statistics for school-related sources of variance. Finally, as data collection for this cross-sectional study occurred annually from 2011 to 2019, secular trends are another source of variance in scores that must be taken into account. Here we expected that the results will be in line with recent original research and meta-analyses and show a decline in cardiorespiratory endurance and an increase in speed^20–22^.

In summary, the hypotheses about age- and sex-related differences in physical fitness tests were tested with a linear mixed model (LMM) that afforded the simultaneous consideration of children, schools, and cohorts as random factors and the estimation of variance components and correlation parameters for (a) test scores, (b) effects of contrasts between the tests, and (c) in the case of schools and cohorts also effects of age-related gains and sex differences. These model parameters serve primarily as measures of statistical control for the fixed effect estimates but may also yield substantive insights about the dynamics of development. For example, for correlations of scores, we expected the usual positive manifold between the tests, but for correlations of effects (i.e., the contrasts between tests) no directed hypotheses were formulated given the usually low reliability associated with difference scores. Although age and sex are between-child factors, they are also both within-school and within-cohort factors, providing us with the opportunity to detect reliable variance components and correlation parameters for these factors. No directed hypotheses were formulated for schools. However, secular trends are well documented, and we expected to replicate them.

## Results

### Overview

Table 1 displays statistics for fixed effects of age (linear) and sex as well as their interactions with the four test contrasts for LMM *m2*. Test-specific z-transformations eliminated main effects of contrasts H1 to H4 (all z ≤ 0.99). Neither the age x sex interaction nor any of the interactions of this term with the four test contrasts were significant (all |*z*|≤ 1.74). Adding quadratic trends of age and their associated interactions to the model, did not significantly contribute to goodness of fit; χ^2^(10) = 9.17, *p* = 0.52. None of the age x sex interaction was significant when tested separately for the five tests (all |*z*|≤ 1.04; see Supplement A for details about both control LMMs).

**Table 1:** Fixed-effect estimates of linear mixed model.

Source of variance	Fixed-effect estimates	Standard error	z-values	Pr ( >|z|)
**Main effects**				
Grand mean (intercept)	− 0.038	0.011	− 3.57*	< 0.001
H1: coordination vs. endurance	0.019	0.024	0.76	0.445
H2: speed vs. coordination	− 0.033	0.033	− 1.01	0.313
H3: powerLOW vs. speed	0.033	0.033	0.99	0.323
H4: powerUP vs. powerLOW	0.005	0.020	0.24	0.808
Age (linear)	0.271	0.009	31.60*	< 0.001
Sex	0.413	0.005	86.52*	< 0.001
Age (linear) x Sex	0.002	0.014	0.14	0.888
**Age** (**linear**) **x Component**				
H1: coordination vs. endurance	0.216	0.012	18.44*	< 0.001
H2: speed vs. coordination	− 0.069	0.011	− 6.22*	< 0.001
H3: powerLOW vs. speed	− 0.007	0.010	− 0.69	0.491
H4: powerUP vs. powerLOW	0.307	0.012	25.87*	< 0.001
**Sex x Component**				
H1: coordination vs. endurance	− 0.260	0.007	− 39.03*	< 0.001
H2: speed vs. coordination	0.078	0.006	12.35*	< 0.001
H3: powerLOW vs. speed	0.066	0.006	11.24*	< 0.001
H4: powerUP vs. powerLOW	0.296	0.007	43.84*	< 0.001
**Age** (**linear**) **x Sex x Component**				
H1: coordination vs. endurance	0.0393	0.023	1.74	0.082
H2: speed vs. coordination	− 0.014	0.021	− 0.63	0.527
H3: powerLOW vs. speed	− 0.020	0.020	− 0.99	0.324
H4: powerUP vs. powerLOW	0.025	0.023	1.07	0.282

H1 to H4 = hypothesis 1–4, endurance = cardiorespiratory endurance (i.e., 6 min run test), coordination = star run test, speed = 20-m linear sprint test, powerLOW = power of lower limbs (i.e., standing long jump test), powerUP = power of upper limbs (i.e., ball push test), * = z-value > 3.0, linear mixed model random factors: cohorts (9), schools (515), children (108,295), observations = 525,126 (missing = 3%). For estimates of variance components and correlation parameters see Table 3.

### Age-related gains

**Figure 2:**
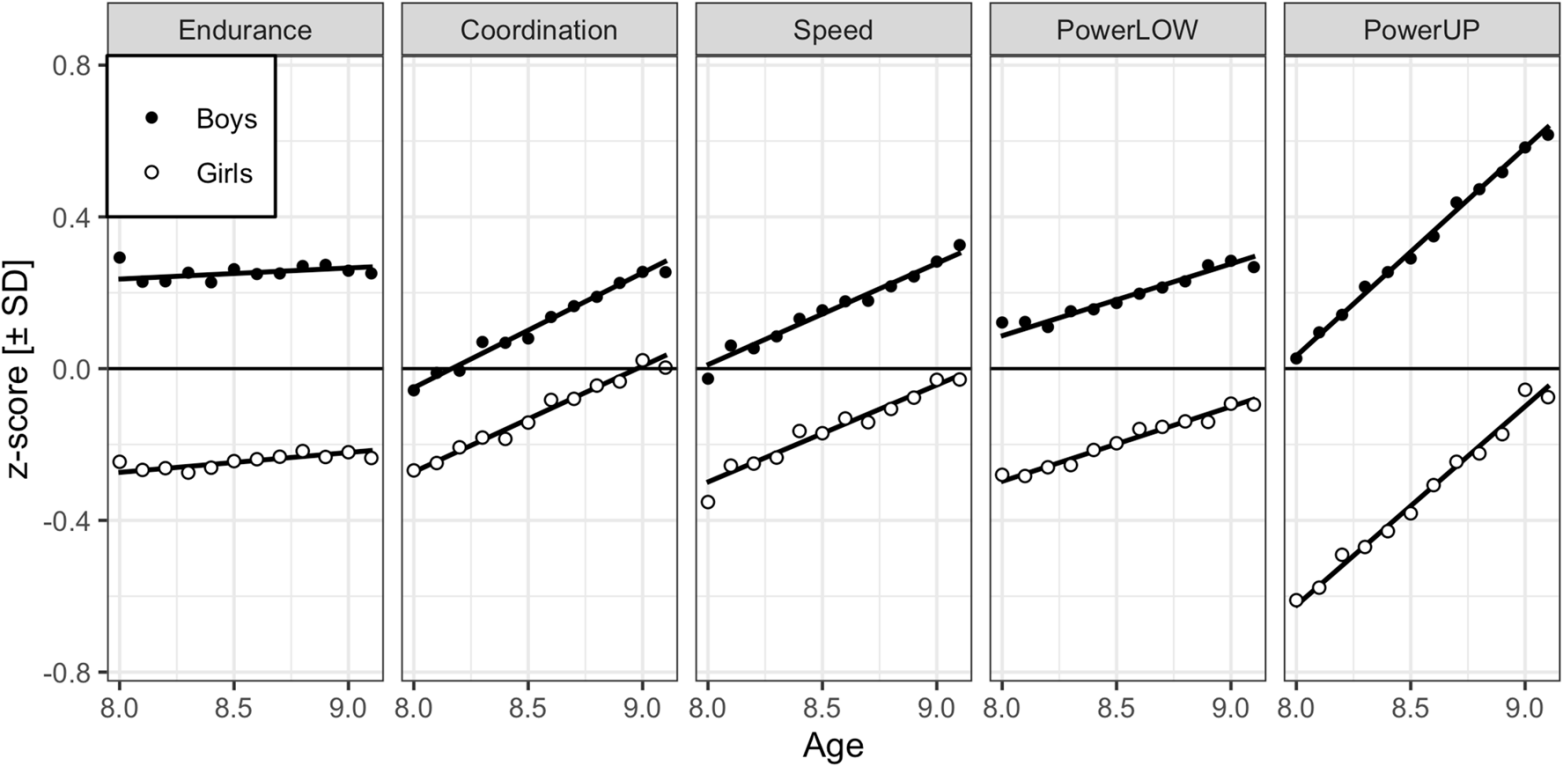
Performance differences between 8.0 und 9.0 years by sex in the five physical fitness tests presented as z-transformed data computed separately for each test. Endurance = cardiorespiratory endurance (i.e., 6 min run test), Coordination = star run test, Speed = 20-m linear sprint test, PowerLOW = power of lower limbs (i.e., standing long jump test), PowerUP = power of upper limbs (i.e., ball push test), SD = standard deviation. Points are binned observed child means; lines are simple regression fits to the observations; 95% confidence intervals for means ≈ 0.05 are not visible.

Figure 2 displays both the gains in physical fitness with age and boys’ higher scores than girls’ in each of the five physical fitness tests. The parallel lines in each panel also visualize the lack of significant evidence for age x sex as well as age x sex x test interactions.

Despite the small age range, the differences were large and visible for all five physical fitness tests; the overall linear trend for age was significant (z = 31.60). The LMM tested the interactions of age with the four test contrasts, that is whether slopes in neighbouring panels (averaged across sex) were parallel. The left two panels (H1) and the right two panels (H4) show that this is clearly not the case—even without a statistical test. Indeed, three of four expected interactions were significant (see second block of Table 1): the age slope was larger for coordination than cardiorespiratory endurance (H1; z = 18.44), larger for coordination than speed (H2; z =  − 6.22), and larger for powerUP than powerLOW (H4; z = 25.87). The difference between age slopes for powerLOW and speed (H3) was not significant (z =  − 0.69).

Counter to this profile of differences, Fig. 2 which is based on observed scores suggests that speed gain is larger than powerLOW gain. Indeed, this contrast was significant for LMM *m1*, that is as long as VCs for cohort-related differences between tests were not in the LMM. The large differences in secular trends are shown in Fig. 4 (below); adjusting for them in the LMM yielded the reported partial effects.

### Sex-related effects

The difference between lines in Fig. 2 displays the expected differences between boys and girls for the performance in the five physical fitness tests; the overall sex effect was significant with z = 86.52. The third block of Table 1 lists statistics for the interactions between sex and the tests contrasts. All interaction terms were significant and in agreement with a priori expectations. Boys performed better than girls on cardiorespiratory endurance than coordination (H1; z =  − 39.03), better on speed than coordination (H2; z = 12.35), better on powerLOW than speed (H3; z = 11.24), and better on powerUP than powerLOW (H4; z = 43.84). The magnitude of these interactions is shown in Fig. 2 in the differences between the parallel lines for boys and girls for neighbouring panels (averaged over age).

### Variance components and correlation parameters

#### Test scores

Table 2 lists estimates of VCs and CPs for the five test scores from a re-parameterized version of LMM `m2` with the same goodness of fit and the same estimates for fixed-effects. The test-related VCs were large for children (0.72–0.78), of medium-size for schools (0.24–0.37), and small for cohorts (0.03–0.06). VCs for the age-related gains (slopes 0.10) and the sex effect (0.05) were also small for schools. It is noteworthy that the differences between schools in the age-related gain of their children is larger than the differences between cohorts.

**Table 2:** Variance components, correlation parameters, and zero-order correlations for test scores.

Component	VC				CP\r			
		End	Coord	Speed	PowerLOW	PowerUP	Age	Sex
**Child**								
Endurance	0.74	1.00	***0.37***	***0.41***	***0.42***	*0.23*		
Coordination	0.76	**0.56**	1.00	***0.44***	***0.44***	*0.32*		
Speed	0.76	**0.61**	**0.67**	1.00	***0.52***	*0.32*		
PowerLOW	0.78	**0.59**	**0.65**	**0.77**	1.00	*0.37*		
PowerUP	0.72	0.25	0.45	0.43	0.50	1.00		
**School**								
Endurance	0.32	1.00	***0.33***	***0.33***	*0.38*	*0.17*	*0.15*	*0.05*
Coordination	0.37	**0.35**	1.00	***0.32***	*0.37*	*0.25*	*0.14*	*0.03*
Speed	0.31	**0.34**	**0.33**	1.00	*0.46*	*0.29*	*0.10*	*0.06*
PowerLOW	0.25	**0.37**	**0.39**	**0.43**	1.00	*0.30*	*0.12*	*0.06*
PowerUP	0.24	0.19	0.18	0.28	0.26	1.00	*0.13*	*−0.04*
Age	0.10	0.44	0.34	0.28	0.32	0.21	1.00	0.10
Sex	0.05	0.12	*−*0.05	0.11	0.19	*−*0.04	0.25	1.00
**Cohort**								
Endurance	0.05							
Coordination	0.04							
Speed	0.06							
PowerLOW	0.04							
PowerUP	0.03							

End = cardiorespiratory endurance (i.e., 6 min run test), Coord = star run test, Speed = 20-m linear sprint test, PowerLOW = power of lower limbs (i.e., standing long jump test), PowerUP = power of upper limbs (i.e., ball push test), VC = variance component, CP \ r = correlation parameter\zero-order correlation; linear mixed model correlation parameters are shown in upright and corresponding pairwise zero-order correlations are shown in italics for children (top) and schools (middle). Theoretically relevant correlations discussed in the text are set in bold. VC for Residual = 0.53. VCs and CPs are based on full set of data; ZOC correlations are based on subsets of data (Child: 96,529 children from 512 schools; School: 93,661 children from 421 schools).

In the first block of Table 2, CPs between tests scores for children are shown in black and the corresponding zero-order correlations (ZOCs) are shown in light grey. The ZOCs are based on 96,529 children with complete test scores; they came from 512 different schools. In the second block, we list corresponding results for schools. For this analysis, we also added the criterion that a school had to report complete data from more than 30 boys and 30 girls to ensure stable estimation of age and sex effects within schools. This criterion left us with 421 schools, 93,661 children, and 468,305 scores.

There were three noteworthy patterns of results. First, as expected, all child- and school-related CPs and ZOCs between test scores were positive. Thus, the five tests represent a latent construct “physical fitness” both for differences between children and for differences between schools. This was also supported by a random-effects principal component analyses (rePCA) of the two orthogonal random-effect structures. The first principal component (PC1) loadings ranged from 0.49 to 0.35 for the child-related PC1 and from 0.45 to 0.29 for the school-related PC1, accounting for 65% and 38% of the respective variances (see Supplement A for details).

Second, the tests did not correlate equally highly with each other. Most notably, the child-related CPs of cardiorespiratory endurance, coordination, speed, and powerLOW correlated very highly between 0.56 and 0.77, but their correlations with powerUP were distinctly smaller (CPs: 0.25–0.50). This observation holds also for the other three correlation matrices, but overall correlations were smaller (see Table 2 for all CPs).

Again, this interpretation was supported by rePCAs (see Supplement A for details). The smallest loading on child- and school-related PC1s was obtained for powerUP (0.35, 0.29). Moreover, for children the loadings for the second PC2s (15%) represented the difference between cardiorespiratory endurance (0.53) and powerUP (− 0.84). Similarly, for schools the third PC3 (12%) represented the difference between the average of cardiorespiratory endurance (0.31) and coordination (0.47) and powerUP (− 0.71). We take this PC2/PC3-based difference score as support for the hypothesis that powerUP favoured heavier children for who strength of arms may mask reduced cardiorespiratory endurance and coordination (see Discussion).

Third, child-related CPs (Table 2, upright numbers) were larger than child-based ZOCs (Table 2, *numbers*). This was a rather striking pattern because one might expect the opposite given that ZOCs were confounded with large effects of age and sex. Conversely, CPs were larger despite adjustment for sex and age differences in the fixed effects and for differences due to schools and cohorts in the random-effect structure of the LMM. The reason for the result is LMM-based shrinkage of conditional means of the units of the random factors in the direction of the GM. Thus, the entire data set was used to “correct” unreliable observations, also called “borrowing strength” to improve predictions. Due to this shrinkage correction for unreliability, CPs revealed the latent relations between measures much more clearly than ZOCs.

#### Effects of test contrasts

In the random-effect structure of LMM *m2*, estimates were returned for child-, school-, and cohort related VCs for GM and the four test contrasts; VCs of age and sex were also estimated for school. CPs for child and school reflect correlations between the contrasts (i.e., effect correlations). The results are shown in Table 3. As in Table 2, CPs are shown in upright numbers and corresponding ZOCs are italics.

**Table 3:** Variance components, correlation parameters, and zero-order correlations for test-related contrasts.

Effects	VC			CP\r					
		GM	H1		H2	H3	H4	Age	Sex
**Child**									
Grand Mean	0.60	1.00	*0.03*		*0.03*	*0.02*	* − ****0.13***		
H1: coordination vs. endurance	0.67	0.11	1.00		* − 0.51*	* − 0.01*	*0.05*		
H2: speed vs. coordination	0.58	0.06	− 0.51		1.00	* − 0.46*	* − 0.07*		
H3: powerLOW vs. speed	0.47	0.04	0.01		− 0.36	1.00	* − 0.40*		
H4: powerUP vs. powerLOW	0.72	− **0.31**	0.15		− 0.20	− 0.23	1.00		
**School**									
Grand Mean	0.20	1.00	*0.08*		* − 0.06*	* − 0.11*	* − 0.19*	***0.19***	*0.05*
H1: coordination vs. endurance	0.40	0.14	1.00		* − 0.55*	*0.00*	*0.05*	*0.00*	* − 0.01*
H2: speed vs. coordination	0.40	− 0.14	− 0.59		1.00	* − 0.50*	* − 0.02*	* − 0.05*	*0.02*
H3: powerLOW vs. speed	0.30	− 0.13	0.02		− 0.50	1.00	* − 0.38*	*0.00*	* − 0.00*
H4: powerUP vs. powerLOW	0.30	− 0.19	− 0.04		0.06	− 0.38	1.00	*0.00*	* − 0.09*
Age (linear)	0.10	**0.48**	− 0.03		− 0.11	− 0.01	− 0.10	1.00	*0.10*
Sex	0.05	0.09	− 0.14		0.13	0.05	− 0.19	0.25	1.00
**Cohort**									
Grand Mean	0.02								
H1: coordination vs. endurance	0.05								
H2: speed vs. coordination	0.08								
H3: powerLOW vs. speed	0.09								
H4: powerUP vs. powerLOW	0.04								

H1 to H4 = hypothesis 1–4, endurance = cardiorespiratory endurance (i.e., 6 min run test), coordination = star run test, speed = 20-m linear sprint test, powerLOW = power of lower limbs (i.e., standing long jump test), powerUP = power of upper limbs (i.e., ball push test), VC = sqrt (variance component), CP\r = correlation parameter\zero-order correlation; linear mixed model correlation parameters are shown in upright and corresponding pairwise zero-order correlations in italics for children (top) and schools (middle). Theoretically relevant correlations discussed in the text are set in **bold**. VC for Residual = 0.54. VCs and CPs are based on full set of data; ZOC correlations are based on subsets of data (Child: 96,529 children from 512 schools; School: 93,661 children from 421 schools).

VCs for test contrasts were larger (0.47–0.72) for children and somewhat smaller, but still highly reliable (0.30–0.40) for schools, especially when compared to VCs estimated for school-related age (0.10) and sex (0.05) effects, and especially when compared to cohort-related effects (0.04–0.09). CPs and ZOCs of effects are smaller than CPs and ZOCs based on test scores because, with the exception of those involving GM, they are all based on difference scores.

There were two results of theoretical relevance. First, there was a negative CP for r_GM.H4_ for children (− 0.31): If we invert the difference score to convert to a positive correlation, then large values of GM correspond to a large difference between powerLOW and powerUP. In other words, the larger powerLOW relative to powerUP, the larger is the expected value for GM. Thus, in line with the special status of powerUP reported above, powerUP is better thought of as an adjustment or sharpening of physical fitness as indicated by PowerLOW than as a genuine indicator of physical fitness by itself. The corresponding ZOC was only − 0.13.

Second, we note three large negative CPs (r H1.H2, r H2.H3, and r H3.H4). However, these correlations are ambiguous because the contrasts had a test in common (i.e., coordination is part of H1 and H2; speed is part of H2 and H3; powerLOW is part of H3 and H4).

**Figure 3:**
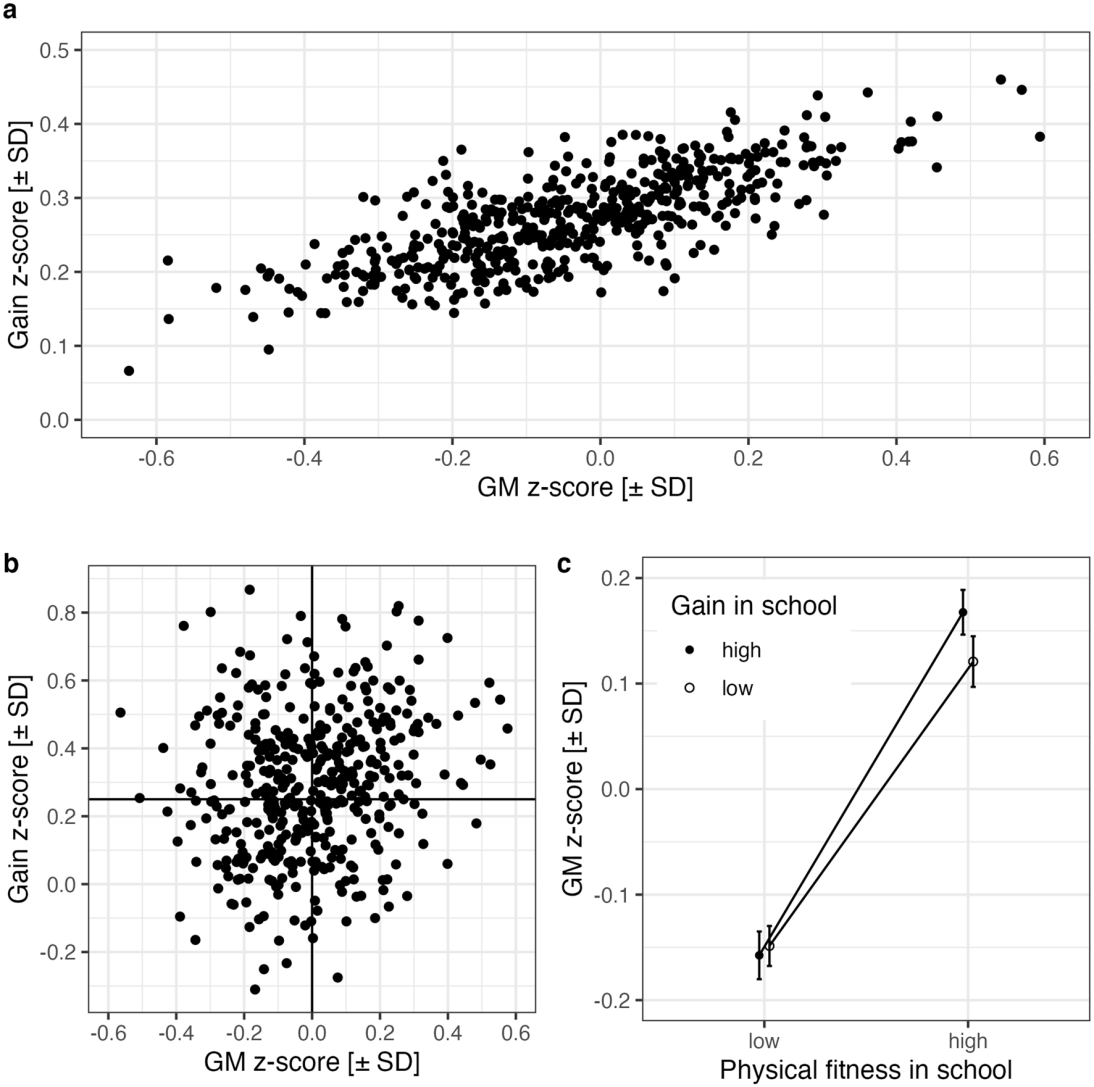
(**a**) Visualization of correlation parameter between Grand Mean (GM) and yearly gains (age effect) of 515 schools using conditional means resulting from shrinkage correction of observed data with LMM parameters. (**b**) Scatterplot of observed GMs and yearly gains (within-school age slopes) for 421 schools reporting data from more than 30 boys and 30 girls. (**c**) After sorting schools into groups of high vs. low physical fitness (split at 0) and high vs. small gain-rate (split at the gain of + 0.25), the interaction corresponding to the CP becomes visible; error bars are 95% CIs. SD = standard deviation.

Third, the largest effect CP was observed between age and GM for schools (+ 0.48) suggesting that “fitter” schools promote more developmental change across the school year. Figure 3A displays a visualization of the CP using a scatterplot of the conditional means of age-related gain over conditional means of GMs of physical fitness for the 515 schools.

The scatterplot is not identical with the CP, in fact the correlation is 0.75, because conditional means are “predictions” of the school age effect and GM using the school data and all model parameters to correct for unreliability in the scores. Indeed, as shown in Fig. 3B, there is no evidence for this relation in the uncorrected scatterplot corresponding to the ZOC of 0.19. A significant CP corresponds to a simple interaction in the data and this interaction can be visualized by sorting schools into those of high and low physical fitness (split at z-score = 0) and those with a high and small gain for their children in the third grade (split at the age slope of + 0.25). In an ANOVA of schools’ observed mean physical fitness values the interaction between these two *post-hoc* grouping factors was significant; F(1, 417) = 4.22, MSe = 0.012, p < 0.05. Of course, from a correlation we cannot infer the direction of causality. “Fitter schools” (e.g., schools offering extracurricular sport-related activities) may facilitate gains in children’s fitness. Alternatively, if we assume that fitter children gain more in a year, then a school’s high fitness as well as the associated large gain could be the result of being attended by fitter children (e.g., due to the school’s location in a high-SES region).

### Cohort-related variance components

**Figure 4:**
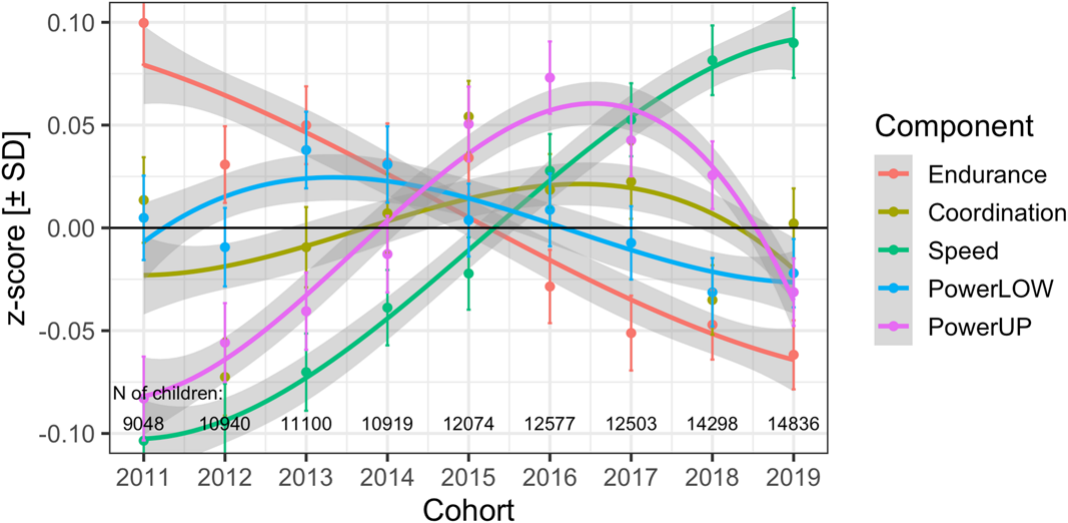
Cohort-related change of components of physical fitness. Points are observed means with 95% CIs. Lines are third-order polynomial trends fitted to children’s scores along with 95% error. Note the much smaller range of the y-axis (i.e., from − 0.10 to + 0.10) compared to age effects shown in Fig. 2. Endurance = cardiorespiratory endurance (i.e., 6 min run test), Coordination = star run test, Speed = 20-m linear sprint test, PowerLOW = power of lower limbs (i.e., standing long jump test), PowerUP = power of upper limbs (i.e., ball push test).

The small, but significant VCs related to the random factor cohort indicate that there were reliable test x cohort interactions; they are shown in Fig. 4. Across the nine years from 2011 to 2019 there was a performance decline for cardiorespiratory endurance and an increase for speed. The other three components exhibit an initial increase followed by a decline of performance in recent years.

### Goodness-of-fit statistics and model residuals

Additional details about the LMM analyses are documented in Supplement A which also contains information about the control LMMs. Despite their complexity, all models converged without problems and there was no evidence of estimates of parameters at their boundaries. Overparameterization was observed only for the most complex LMM *m4.* Thus, with this exception, the LMMs were supported by the data. Finally, we carried out residual-based diagnostics (e.g., q-q plot, standardized residuals over fitted values, etc.) for the reference LMM *m2* with CPs for effects (Table 1, Table 3). These tests did not reveal any problems.

## Discussion

The aim of this study was to examine *short-term ontogenetic cross-sectional* developmental differences in physical fitness for five tests tapping *health*- and *skill-related* components of physical fitness in a large sample of 108,295 German eight-year old children. Even in a single prepubertal year of life (1) performance increases linearly with chronological age in all physical fitness tests, (2) boys outperform girls in all physical fitness tests with sex differences being larger for tests requiring muscle mass and being smaller for tests requiring motor coordination, (3) the tests differ, mostly as expected, in the size of age and sex effects, (4) four of the five tests represent a common construct (i.e., correlate strongly positively with each other)—the exception is the ball push test that requires powerUP, (5) “physically fit schools” apparently promote more developmental gains within a year (but this is only correlational evidence) and (6) diverging secular trends for cardiorespiratory endurance (negative) and speed (positive) are in agreement with other research and meta-analyses. Furthermore, there was no evidence for an interaction of age and sex—with each other or with the test contrasts despite an abundance of statistical power. Counter to folklore, a very large data set (i.e., 108,295 subjects and 525,126 observations) did not “automatically” render everything significant! Given this statistical power, we are strongly inclined to interpret the absence of evidence for interactions as evidence of absence of interactions for these five tests of physical fitness^16^.

Boys outperformed girls in all four physical fitness components (cardiorespiratory endurance, coordination, speed, power [LOW/UP]). This is in line with other studies reporting normative values^2–5,14,23–26^. For instance, Tambalis et al.^5^ reported that boys aged 6–18 years showed significantly better performances for cardiorespiratory endurance (i.e., 20 m shuttle run test), powerLOW (i.e., standing long jump test), and agility (i.e., 10 × 5 m agility shuttle run test) compared to girls. Furthermore, De Miguel-Etayo et al.^3^ described that boys aged 6–10 years significantly outperformed girls in cardiorespiratory endurance (i.e., 20 m shuttle run test), speed (i.e., 40 m sprint test), and powerLOW (i.e., standing long jump test).

What are the reasons for the observed large test-specific sex differences? Sex-related differences in body composition appear to be likely candidates to account for the observed findings. The larger sex effect in powerUP compared to powerLOW can be explained by a better proportion of strength relative to body mass especially in the upper limbs in boys compared to girls^27,28^. The large difference in cardiorespiratory endurance can be explained by physiological factors such as boys’ better mechanical efficiency and fractional utilisation of oxygen^2,29^. Furthermore, muscle mass^1^ and muscle cross-sectional area^13^ favour boys especially in physical fitness tests that recruit muscle mass. Beside these anthropometric factors and physiological demands, sociocultural aspects may also explain the sex difference. Haywood and Getchell^30^ reported that girls usually participate in sports that require balance and flexibility (e.g., gymnastics, figure skating) compared to boys who rather participate in strength-related activities.

The sex effect was also significantly stronger for powerLOW than for speed. PowerLOW is determined much more by muscle mass where boys usually outperform girls^2,3,5^. In contrast, speed is less influenced by muscle mass than by motor coordination where sex differences are comparatively small^31^ or were not found at all^17^. Therefore, the sex effect in powerLOW might be larger than in speed. Obviously, the demand of coordination relative to power and cardiorespiratory endurance is even larger in the star run test than in the 20-m linear sprint test^32–34^ and this could be a reason why the sex effect is smaller for the star run test than for speed.

The observed decrease of sex differences in performances with increased demands for coordination seems to be plausible. Coordination draws on many different central (e.g., brain) and peripheral sites (e.g., motor units) within the nervous system^35^. The more a test engages the brain, the less relevant sex is a performance limiting factor. In summary, the decrease of size of sex effects across tests can most likely be explained by a decrease in the relevance of muscle mass (favouring boys over girls)^1^ and an increase in the relevance in motor coordination (associated with small or no sex effects)^17,31^.

Within their ninth year of life, older children significantly outperform younger ones in all five fitness tests. This was expected^3,5,14^, but there were no significant gains for a sample of 240 children in the tenth year of life (see insets of Fig. 1). The absence of evidence for nonlinear relations between performance and chronological age as well as the similarity of gains for boys and girls are quite remarkable (see Fig. 2), especially in light of what *long-term ontogenetic longitudinal* research reveals when children are a few years older. Obviously, the current test battery does not detect the onset of puberty in the ninth year of life! Nevertheless, the different age-related grains for tests are compatible with what is known about the development of basic physiological parameters: Growth of body mass and height positively influence the performance in *skill-related* physical fitness components of coordination, speed and power. In contrast, increases in body mass have a negative impact on performance in tests such as cardiorespiratory endurance (e.g.., 6 min run test) where the own body mass has to be accelerated continuously. The smaller age effect for cardiorespiratory endurance compared to *skill-related* components to the remaining physical fitness components is in line with a study conducted by de Miguel-Etayo et al.^3^. The authors could not find significant age differences in cardiorespiratory endurance (i.e., 20 m shuttle run test) but amongst other in powerLOW (i.e., standing long jump test) and strength (i.e., absolute handgrip strength test) in a sample of children aged 6–10 years. Similar, Viru et al.^36^ stated that an accelerated improvement in cardiorespiratory endurance occurs at the ages of 11–15 years in boys and 11–13 years in girls.

As far as the differential age effects between the components of physical fitness are concerned, tests of coordination, speed, and powerLOW share the highest correlations among the five tests. These three tests share the relevance of muscle mass yielding power, but they differ in the relevance of coordination. As mentioned above, the sex effect within these three tests (i.e., star run test < 20-m linear sprint test < standing long jump test; see differences between lines in Fig. 2) is in line with their ranking on coordination. For age-related gains within the school year, the partial effects yielded star run test > 20-m linear sprint test and standing long jump test, corresponding (roughly) to their ranking on motor coordination. The special status of powerUP (i.e., ball push test) is not only evident with respect to its lower correlations with other tests, but also with respect to the size of the age effect—by far the largest of the five tests (see Fig. 2).

Obviously, the performance in powerUP was influenced by factors other than physical fitness. We propose that body mass contributes to performance in tests that assess powerUP. Heavier children usually have a higher muscle mass compared to normal weighted children^37^. While a higher muscle mass positively influences performances in non-weight-bearing tests (e.g., ball push test), it negatively influences performances in weight-bearing tests (e.g., standing long jump test) because the body mass has to be accelerated in contrast to non-weight-bearing tests^12^. Therefore, tests for the assessment of powerUP favour heavier children for whom strength of arms and trunk may mask reduced cardiorespiratory endurance and coordination. Thus, a high score for powerUP may be more indicative of a lack of overall physical fitness because it does not measure physical fitness to the same degree than the other four tests (i.e., small correlations with the other four tests and GM). Our results suggest that the best indicator of physical fitness is the average of the first four tests (cardiorespiratory endurance, coordination, speed, and powerLOW). Analyses including body mass (not measured in the present study) could support the interpretation of the special status powerUP in the assessment of physical fitness.

The linear mixed model included school as a second random factor, supported the estimation of correlation parameters for test scores and age effects, and revealed a strong correlation (r = 0.48) between the age effect and overall physical fitness. The direction of causality is not clear, but in line with Hattie^19^ and García-Hermoso et al.^38^. The results are in agreement with the hypothesis that schools differ in how much they qualitatively promote the development of children’s physical fitness^38^. The development of physical fitness depends on the quality of the physical education lessons and especially on the quality of the physical education teacher teaching physical education^38^. These school differences in the age effect of physical fitness within the third grade were strong enough to yield reliable differences in the overall physical fitness of their children.

Our study is not without limitations. First, the five tests do not cover all components of physical fitness^18^ and there are alternative tests for each component. Although our results do not necessarily generalize to other components like muscle strength, muscle endurance, or balance^18^, they represent those for whom an early detection of puberty was most likely. Second, from a cross-sectional study we cannot know whether the linear gains for tests hold at the individual level or are the result of averaging over individual differences in non-linear growth curves. Obviously, high-density monitoring within a year would be desirable, but such longitudinal data are not without their own problems. For example, learning effects could be reduced due to the completion of at least three familiarization sessions^39^ to separate learning effects from growth. However, motivational factors may also play a role. The longitudinal cardiorespiratory endurance data in Fig. 1 suggest no further growth or even a decline in performance for 12 year old children^6^. This is obviously not in agreement with what we know about the objective development of cardiorespiratory endurance^2,4,5^. Third, divergence between cross-sectional and longitudinal profiles is usually cultural change (i.e., cohort effects). Data were accumulated from 2011 to 2019, which is long enough for cohort effects to materialize. The variance of between cohort differences in physical fitness was by far the smallest source examined in this study. Fourth, several important covariates (e.g., physical activity, sedentary behaviour, socioeconomic indicators) which affect physical fitness were not assessed but could provide an additional insight into the development of physical fitness. Fifth, physical fitness is highly related to biological maturity and more mature youth outperform less mature youth in physical fitness^40^. Our study indicates the strongest test of an early detection of puberty-related divergence with physical-fitness tests that we are aware of. We conclude that puberty-related development has not started or is not strong enough yet in eight-year old children to be picked up with physical fitness or some of its components. A joint analysis with anthropometric measures (i.e., body mass, body height) and status of biological maturity (e.g., peak height velocity, secondary sex characteristics) will allow a stronger test of the hypothesis that the onset of puberty can already be detected in the ninth year of life.

To sum up, the *short-term ontogenetic* results of this cross-sectional study revealed test-specific age and sex effects, but no interaction between age and sex despite an abundance of statistical power. According to Ortega et al.^14^ physical fitness data of an individual should be compared with reference values of a sex and age-matched similar general population. Such norms are usually only available on annual or semi-annual basis. Our results suggest that physical education teachers, coaches, or researchers can use a proportional adjustment to adequately evaluate physical fitness of prepubertal school-aged children. Furthermore, especially muscle strength / powerUP should be promoted in sport activities for girls in order to reduce the large sex difference between boys and girls. Lastly, physical education teachers, coaches, or researchers should be careful in the interpretation of the ball push test and should consider that it does not measure physical fitness to the same degree than the other tests.

## Methods

### Sample and study design

This cross-sectional study is part of the EMOTIKON project. The study was mandated and approved by the Ministry of Education, Youth and Sport of the Federal State of Brandenburg, Germany. The Brandenburg School Law^41^ requires that parents are comprehensively informed prior to the start of the study. Consent is not needed given that the tests are obligatory for both, children and schools. Physical fitness tests were administered to *all* third-graders in the state annually between September and November from 2011 to 2019. Physical fitness tests were also administered to 2009 and 2010 cohorts, but later in the school year that is between March and April. Due to the seasonal variation in physical fitness these data were not included. Research was conducted according to the latest Declaration of Helsinki.

We started with data from 144,045 children. Of those, we included only healthy children who had been enrolled within the legal key date of the Federal State of Brandenburg, that is in a given year of school enrolment they were at least 6.00 and at most 6.99 years old on September 30^th^ and, therefore, varied between 8.00 and 8.99 years in the third grade (n = 110,669). In addition to early-entry (n = 2,664), late-entry (n = 30,457) and children without information about birthdate (n = 255), we did not include children with signs of emotional (e.g., autism) and/or physical disorders (e.g., disabilities like infantile cerebral palsy) that were evaluated by the responsible and experienced physical education teacher based on a medical clearance (n = 28). After the first iteration, the LMM-based conditional means of the random effects identified one school as an extreme outlier on several tests across the years and the 171 children of that school were excluded as well. Finally, we applied a  ± 3 SD criterion to individual test scores which led to the exclusion of another 2,175 children (2%). This left us with 108,295 children (i.e., 75% of those tested) from 515 different schools.

### Physical fitness tests

Physical fitness was assessed with the EMOTIKON test battery (www.uni-potsdam.de/en/emotikon/projekt/methodik for further information on the test protocols). The five tests measured cardiorespiratory endurance (i.e., 6 min run test), coordination (i.e., star run test), speed (i.e., 20-m linear sprint test), power of lower limbs (powerLOW [i.e., standing long jump test]), and power of upper limbs (powerUP [i.e., ball push test]). The EMOTIKON test battery officially includes six tests. Up to 2015 the sixth test was the stand and reach test (flexibility) that was then exchanged against the single-leg balance test (balance). Due to the much smaller of number of scores and their confound with cohort these tests were not included in the analyses. The five tests yielded 525,126 scores from the 108,295 children (i.e., 3% missing test scores).

Qualified physical education teachers of each school administered the tests according to standardized test protocols during the regular physical education classes in the participating schools (www.uni-potsdam.de/en/emotikon/projekt/methodik for further information on the test protocols). Teachers were instructed in a standardized assessment through an advanced training. Tests were always conducted in the morning between 8 and 12 o’clock. Encouragement to achieve the best performance was permitted. Prior to testing, all third-graders performed a standardized warm-up program consisting of different running exercises (e.g., side-steps) and small games (e.g., playing tag).

#### Cardiorespiratory endurance

Cardiorespiratory endurance was assessed with the 6 min run test*.* Children had to run as far as they could within six minutes around an official volleyball field (9 × 18 m, every 9 m a pylon/marker was set beside the running court [i.e., six pylons around the field]) at a self-paced velocity. Split time was given every minute. The maximal distance achieved during the six minutes in meters to the nearest nine-meters marker was used as dependent variable in the analysis. The 6 min run test was reliable (test–retest) in children aged 7–11 years with an intraclass correlation coefficient (ICC) of 0.92^42^. The 6 min run test correlated at r = 0.69 (p < 0.01) with V̇O_2max_ assessed via a gas analysis during a progressive treadmill test in children aged 9–11 years^43^.

#### Coordination

**Figure 5:**
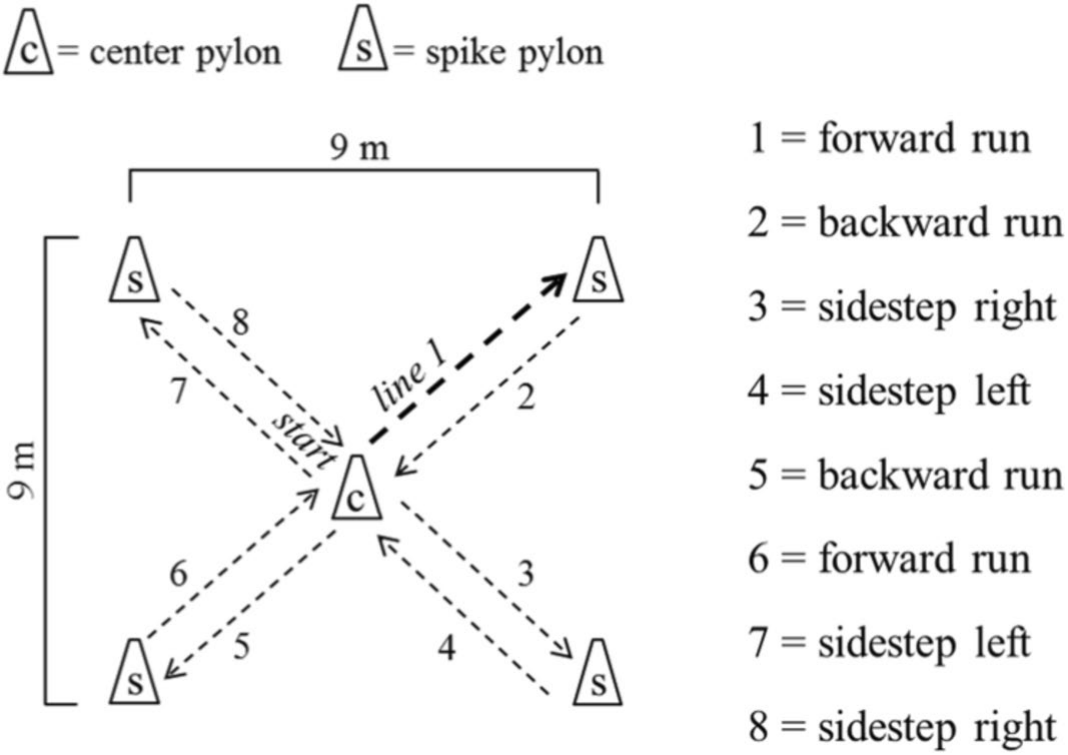
Schematic description of the star run test (adapted from Golle et al. ^6^).

Coordination under time pressure was tested with the star run test (see Fig. 5). Children had to complete a parkour with different movement directions and movement forms (i.e., running forward, running backward, side-steps to the left side, side-steps to the right side). The parkour had to be performed in a given order over a 9 × 9 m star-shaped area where each of the four spikes is marked by a pylon. After starting in the centre of the star, children had to complete the parkour as fast as they could by running in every movement form two times within the given sequence. They had to touch each pylon with the hand. The whole covered distance is 50.912 m. The faster of two test trials was used in the analysis. The shortest time for completing the parkour in seconds to the nearest 1/10 s was measured using a stopwatch and was used as dependent variable in the analysis. The star run test was reliable (test–retest) in 8–10 year old children with an ICC of 0.68^44^.

#### Speed

Speed was assessed with the 20-m linear sprint test. After an acoustic signal, the children had to sprint out of a frontal erect posture as fast as they could over a distance of 20 m for two times; the faster test trial was used in the analysis. The shortest time for sprinting the 20 m in seconds to the nearest 1/10 s was measured using a stopwatch and was used as dependent variable in the analysis. The 20-m linear sprint test was reliable (test–retest) in children aged 7 to 11 years with an ICC of 0.90^42^.

#### Power of lower limbs (PowerLOW)

PowerLOW was tested using the standing long jump test. Out of a standing frontal posture the children had to jump as far as they could. The participants had to land with both feet together. They were allowed to swing their arms prior to and during the jump, but after landing the hands were not allowed to touch the floor. The distance in meters to the nearest one centimeter between toes at take-off and heels at landing was determined using a measuring tape; the better of two test trials was used in the analysis. The standing long jump test was reliable (test–retest) in children aged 6–12 years with an ICC of 0.94^45^.

#### Power of upper limbs (PowerUP)

PowerUP was assessed through the ball push test. From a standing position the children had to push a 1 kg medicine ball starting in front of the chest with both hands as far as they could for two times; the better of two test trials of longest pushing distance was used in the analysis. The maximal ball push distance in meters to the nearest ten centimeters was determined with a measuring tape and used as dependent variable in the analysis. The ball push test was reliable (test–retest) in children aged 8–10 years with an ICC of 0.81^44^.

### Statistics

Pre- and post-processing of data were carried out in the R environment of statistical computing^46^ using the *tidyverse* package^47^. For measures of cardiorespiratory endurance (i.e., 6 min run test), powerLOW (i.e., standing long jump test) and powerUP (i.e., ball push test), higher scores indicated better physical fitness. For measures of coordination (i.e., star run test) and speed (i.e., 20-m linear sprint test), a Box-Cox distributional analyses indicated that a reciprocal transformation brought scores in line with the assumption of a normal distribution^48^. Therefore, we converted scores from seconds to meters/seconds (i.e., pace scores; star run test = 50.912 [m] / time [s]; 20-m linear sprint test = 20 [m] / time [s]). These transformations also had the advantage that a large value was indicative of a good physical fitness for all five measures.

For each test, we determined the ± 3 SD boundary separately for boys and girls. Measurement outside these boundaries were usually implausible (i.e., recording errors) or extreme outliers. They were treated as missing values (3%). Finally, we converted scores within tests (aggregated over boys and girls) to z-scores to facilitate comparison of test, age and sex effects.

Statistical inference was based on a linear mixed model (LMM) estimated with the *MixedModels* package^49^ in the *Julia* programming language^50^. The LMM included child (N = 108,295), school (N = 515), and cohort (N = 9) as three random factors; the total number of observations (i.e., max = 5 per child) was 525,126. Fitted model objects were processed with random-effects principal component analysis to obtain loadings of the variance–covariance matrix of the random effects and facilitate its interpretation.

As fixed effects, we specified four sequential-difference contrasts for the five tests: (H1) coordination vs. cardiorespiratory endurance, (H2) speed vs. coordination, (H3) powerLOW vs. speed, and (H4) powerUP vs. powerLOW. Also included were the effect of age (centered at 8.5 years) as a second-order polynomial trend, the effect of sex (boys–girls), and all interactions between contrasts, age, and sex. Given the large number of observations, children, and schools, we adopted a two-sided z-value > 3.0 as significance criterion for the interpretation of fixed effects.

Child, school, and cohort were included as random factors. With three random factors there was a need for selecting a random-effect structure that included theoretically relevant and reliable variance components (VCs) and correlation parameters (CPs), but was also still supported by the data (i.e., was not overparameterized). Tests varied within children, schools, and cohorts; age and sex varied between children, but within schools and within cohorts. Therefore, in principle, VCs and CPs of linear effects of age and sex could be estimated for schools and cohorts, but not for children.

Parsimonious model selection occurred in two major steps without knowledge or consideration of fixed-effect estimates^51^; details are provided in Supplement A. We started with a model including Grand Mean (varying intercepts) for all three random factors and, given the large numbers of 108,926 children and 515 schools and the small number of nine cohorts, included also test-related VCs and CPs for child and school and age-related and sex-related VCs and CPs for school, but not for cohort. This LMM *m1* was well supported by the data. In the second major step, we increased the complexity of the random-effect structure for cohort by adding test-related VCs (LMM *m2*), then test-related CPs (LMM *m3*), and finally age- and sex-related VCs and CPs (LMM *m4*).

LMM *m4* was not supported by the data (i.e., the fit was singular) and did not significantly improve the goodness of fit over LMM *m3;* delta *χ*^*2*^ (13) = 14.11, *p* = 0.37. LMM *m3* improved the goodness of fit over LMM *m2* according to the likelihood ratio test, *χ*^*2*^ (10) = 48.45, *p* < 0.001, but not when the increase in model complexity is penalized according to LMM *m2* =  0,00012345 and LMM *m3* =  0,00012345). As we had no directed hypotheses relating to test-related CPs for the factor cohort, we stayed with LMM *m2* which represented a very large improvement in goodness of fit relative to LMM *m1*; *χ*^*2*^ (4) = 1489.57, *p* < 0.001. We also estimated LMM *m2* with two alternative parameterizations that did not change the goodness of fit, but yielded information about CPs between test scores instead of test effects (i.e., contrasts). Finally, we fitted two control LMMs to test the significance of quadratic age trends for fixed effects and the absence of evidence for sex x age interactions separately for each fitness component (i.e., nested within the five levels of the factor test).

## Supplementary Information

Supplementary Information.

## Data Availability

The datasets generated and analysed during the current study as well as Julia and R scripts are available in the Open Science Framework (OSF) repository : https://osf.io/2d8rj/.
