# Supplementary material for: Age and sex effects in physical fitness components of 108,295 third graders including 515 primary schools and 9 cohorts
Source: Sci Rep. 2021 Sep 2;11:17566. doi: 10.1038/s41598-021-97000-4 (PMC8413306; doi:10.1038/s41598-021-97000-4)
Supplement: Supplementary file 1 — Supplementary Information. [file 41598_2021_97000_MOESM1_ESM.pdf]

Supplement A: Linear Mixed Models for manuscript: ‘Age and sex effects in physical fitness components of 108,295 third graders including 515 primary schools and 9 cohorts’ written by Fühner, T., Granacher, U., Golle, K., & Kliegl, R.

2021-07-27 (updated: 2021-07-28 10:02:10)

Contents

1 Background **3**

2 Readme for ‘fggk21.rds’ **3**

3 Preprocessing **3**

4 Model formulae **4**

5 Model selection **5**

5.1 LMMs with effects in FE and RES 5

6 LMM with CPs for test effects/contrasts (Table 1 and Table 3) **6**

6.1 LMM `m1` 6

6.2 Effects PCA w/ CPs 7

6.2.1 Random factor `Child` 7

6.2.2 Random factor `School` 7

7 LMM with CPs for Test scores (Table 2) **8**

7.1 LMM `m1L` 8

7.2 Levels PCA w/ CPs 9

7.2.1 Random factor Child 9

7.2.2 Random factor School 10

8 LMM with quadratic trends of age **10**

9 LMM with Age x Sex interactions nested in levels of Test **11**

10 A few standard observation-level residual plots **13**

11 Julia and package versions **14**

12 References **14**

1 Background

This supplement accompanies Fühner, T., Granacher, U., Golle, K., & Kliegl, R. (2021). *Age and sex effects in physical fitness components of 108,295 third graders including 515 primary schools and 9 cohorts.* Scripts and data are available in OSF repository: https://osf.io/2d8rj/

Fitting of linear mixed models for model selection, component analyses of the random-effect structures, and control models as well as model comparisons were carried out with the *MixedModels.jl* package in **Julia** (script: FGGK21.jl on osf repo); table contents and figures were generated in **R** (script: FGGK21.Rmd on osf repo).

2 Readme for 'fggk21.rds'

Number of scores: 525,126

1. Cohort: 9 levels; 2011-2019
2. School: 515 levels
3. Child: 108,295 levels; all children are between 8.00 and 8.99 years old
4. Sex: "Boys" (n= 53,209), "Girls" (n=55,086)
5. age: test date - middle of month of birthdate
6. Test: 5 levels
    - Endurance (`Run`, cardiorespiratory endurance): 6 min run test [m]; to nearest 9m in 9x18m field
    - Coordination (`Star_r`): star run test [m/s]; 9x9m field, 4 x diagonal = 50.912 m
    - Speed(`S20_r`): 20-m linear sprint test [m/s]
    - PowerLOW (`SLJ`, Power of lower limbs): standing long jump test [cm]
    - PowerUP (`BPT`, Power of upper limbs): 1-kg ball push test [m]
7. score - see units

3 Preprocessing

- read data
- center age
- compute indicator for quadratic trend of age
- specify contrasts for test factor

```
using RCall, DataFrames, DataFrameMacros, StatsBase, MixedModels

dat = rcopy(R"readRDS('./data/fggk21.rds')");

describe(dat)
select!(groupby(dat, :Test), :, :score => zscore => :zScore)

@transform!(dat, :a1 = :age - 8.5, :a2 = :a1 ^ 2);

contr = merge(
       Dict(nm => SeqDiffCoding() for nm in (:Test, :Sex)),
       Dict(nm => Grouping() for nm in (:School, :Child, :Cohort)),
   );
```

4 Model formulae

It was apparent that the data would support an LMM with the four test contrasts, linear slope for age, and the sex effect as variance components and correlation parameters for random factors child and school where appropriate. (Age and sex vary between children and cannot be included for this random factor.) Special consideration was given to the structure of the random factor `Cohort` which had only nine "levels". We considered four models `m0` to `m3` of increasing complexity as shown in the following. We also estimated each of these models in two alternative parameterizations as described below (i.e., a total of twelve LMMs).

Here are the model formulae:

```
m0   = "~ 1 + FE + (1+Test | Child) + (1+Test+a1+Sex | School) + (1 | Cohort)"
m1   = "~ 1 + FE + (1+Test | Child) + (1+Test+a1+Sex | School) + zerocorr(1+Test | Cohort)"
m2   = "~ 1 + FE + (1+Test | Child) + (1+Test+a1+Sex | School) + (1+Test | Cohort)"
m3   = "~ 1 + FE + (1+Test | Child) + (1+Test+a1+Sex | School) + (1+Test+a1+Sex | Cohort)"

m0L  = "~ 1 + FE + (0+Test | Child) + (0+Test+a1+Sex | School) + (1 | Cohort)"
m1L  = "~ 1 + FE + (0+Test | Child) + (0+Test+a1+Sex | School) + zerocorr(0+Test | Cohort)"
m2L  = "~ 1 + FE + (0+Test | Child) + (0+Test+a1+Sex | School) + (0+Test | Cohort)"
m3L  = "~ 1 + FE + (0+Test | Child) + (0+Test+a1+Sex | School) + (0+Test+a1+Sex | Cohort)"

m0LL = "~ 0 + FE + (0+Test | Child) + (0+Test+a1+Sex | School) + (1 | Cohort)"
m1LL = "~ 0 + FE + (0+Test | Child) + (0+Test+a1+Sex | School) + zerocorr(0+Test | Cohort)"
m2LL = "~ 0 + FE + (0+Test | Child) + (0+Test+a1+Sex | School) + (0+Test | Cohort)"
m3LL = "~ 0 + FE + (0+Test | Child) + (0+Test+a1+Sex | School) + (0+Test+a1+Sex | Cohort)"
```

LMM `m0` to `m3` represent the default specification with effects both in the fixed-effect (FE) and the random-effect structure (RES) of the models with increasing numbers corresponding to an increase in complexity of the RES of the random factor `Cohort`. We start with a cohort-related variance component (VC) for the Grand Mean (GM) in LMM `m0`. Then, in LMM `m1` we add cohort-related VCs for the contrast effects specified for the factor `Test` and, in LMM `m2`, also estimate cohort-related correlation parameters (CPs). In the most complex LMM `m3`, we add cohort-related components for the linear effect of age (`a1`) and for the effect for `Sex`.

LMM `m0L` to `m3L` differ from the above only in that in the RES we do not estimate VCs and CPs for contrasts specified for the differences between tests (i.e., for test contrasts), but VCs and CPs for the test scores (i.e., we exchange `1` for `0`). Thus, the CPs should reflect the typical positive manifold of correlations that we expect from a test battery that is intended to measure something like a latent construct of *physical fitness*.

LMM `m0LL` to `m3LL` estimate scores not only in the RES, but also in the FE part of the model. Thus, we estimate means for test scores after statistical adjustment for all the other variables. This is not very informative in the present context because all tests were transformed to z-scores prior to analyses, that is their means were expected to be zero anyway.

LMM `m1` and LMM `m1L` are reported in some detail in the paper. In addition, there are two *post-hoc* control LMMs. The first one tests the significance of adding quadratic trends of age (`m1_agesq`) to `m1`; the second one tests the age x Sex interaction for each of the five tests, that is nested within levels of the `Test` factor (`m1LL_nested`).

5 Model selection

- FE: fixed effect part
- RES: random-effect structure

5.1 LMMs with effects in FE and RES

```
show(stdout, MixedModels.likelihoodratiotest(m0, m1, m2, m3))
```

```
Model Formulae
1: zScore ~ 1 + Test + a1 + Sex + Test & a1 + Test & Sex + a1 & Sex + Test & a1 & Sex +
(1 + Test + a1 + Sex | School) + (1 + Test | Child) + (1 | Cohort)
2: zScore ~ 1 + Test + a1 + Sex + Test & a1 + Test & Sex + a1 & Sex + Test & a1 & Sex +
(1 + Test + a1 + Sex | School) + (1 + Test | Child) + MixedModels.ZeroCorr((1 + Test | Cohort))
3: zScore ~ 1 + Test + a1 + Sex + Test & a1 + Test & Sex + a1 & Sex + Test & a1 & Sex +
(1 + Test + a1 + Sex | School) + (1 + Test | Child) + (1 + Test | Cohort)
4: zScore ~ 1 + Test + a1 + Sex + Test & a1 + Test & Sex + a1 & Sex + Test & a1 & Sex +
(1 + Test + a1 + Sex | School) + (1 + Test | Child) + (1 + Test + a1 + Sex | Cohort)

     model-dof     -2 logLik          2   2-dof  P(> 2)

[1]         65  1315560.5115
[2]         69  1314070.9604  1489.5512       4  <1e-99
[3]         79  1314022.4516    48.5087      10  <1e-06
[4]         92  1314008.8153    13.6364      13  0.3999
```

```
mods = [m0, m1, m2, m3];
gof_summary = DataFrame(dof=dof.(mods), deviance=deviance.(mods),
                        AIC = aic.(mods), AICc = aicc.(mods), BIC = bic.(mods))
```

Row	dof	deviance	AIC	AICc	BIC	Selection
m0	65	1.31556e6	1.31569e6	1.31569e6	1.31642e6	-
m1	69	1.31407e6	1.31421e6	1.31421e6	1.31498e6	+, using BIC
m2	79	1.31402e6	1.31418e6	1.31418e6	1.31506e6	-
m3	92	1.31401e6	1.31419e6	1.31419e6	1.31522e6	-

The comparisons of goodness of fit suggested that `m1` and `m2` were supported by the data. BIC suggested to go with `m1` and since we did not have any specific hypotheses relating to cohort-related correlation parameters, we selected LMM `m1`. Goodness-of-fit statistics for reparameterized alternatives also were in line with this selection.

6 LMM with CPs for test effects/contrasts (Table 1 and Table 3)

6.1 LMM m1

```
f1 = @formula zScore ~ 1 + Test*a1*Sex +
     (1+Test+a1+Sex | School) +  (1+Test | Child) + zerocorr(1+Test | Cohort);

m1 = fit(MixedModel, f1, dat, contrasts=contr);
show(stdout, m1)
```

```
Minimizing 5179  Time: 0 Time: 1:00:38 ( 0.70  s/it)
Linear mixed model fit by maximum likelihood
 zScore ~ 1 + Test + a1 + Sex + Test & a1 + Test & Sex + a1 & Sex + Test & a1 & Sex +
 (1 + Test + a1 + Sex | School) + (1 + Test | Child) + MixedModels.ZeroCorr((1 + Test | Cohort))
    logLik     -2 logLik       AIC          AICc           BIC
 -657035.4802 1314070.9604 1314208.9604 1314208.9788 1314979.7865

Variance components:
            Column     Variance  Std.Dev.   Corr.
Child    (Intercept)   0.3578882 0.5982376
         Test: Star_r  0.4532539 0.6732414 +0.11
         Test: S20_r   0.3405327 0.5835518 +0.06 -0.51
         Test: SLJ     0.2222123 0.4713940 +0.04 +0.01 -0.36
         Test: BPT     0.5128780 0.7161550 -0.31 +0.15 -0.20 -0.23
School   (Intercept)   0.0409766 0.2024269
         Test: Star_r  0.1584968 0.3981166 +0.14
         Test: S20_r   0.1600540 0.4000674 -0.14 -0.59
         Test: SLJ     0.0913556 0.3022508 -0.13 +0.02 -0.50
         Test: BPT     0.0899933 0.2999888 -0.19 -0.04 +0.06 -0.38
         a1            0.0093739 0.0968189 +0.48 -0.03 -0.11 -0.01 -0.10
         Sex: Boys     0.0024064 0.0490550 +0.09 -0.14 +0.13 +0.05 -0.19 +0.25
Cohort   (Intercept)   0.0002397 0.0154825
         Test: Star_r  0.0023612 0.0485920   .
         Test: S20_r   0.0068023 0.0824762   .     .
         Test: SLJ     0.0080948 0.0899710   .     .     .
         Test: BPT     0.0016617 0.0407645   .     .     .     .
Residual               0.3197249 0.5654422
 Number of obs: 525126; levels of grouping factors: 108295, 515, 9

  Fixed-effects parameters:

                                     Coef.  Std. Error       z  Pr(>|z|)

(Intercept)                     -0.0383176   0.0107315   -3.57    0.0004
Test: Star_r                     0.0186795   0.0244388    0.76    0.4447
Test: S20_r                     -0.0333225   0.0330374   -1.01    0.3132
Test: SLJ                        0.0327451   0.0331084    0.99    0.3227
Test: BPT                        0.004765    0.0195805    0.24    0.8077
a1                               0.271181    0.00858239  31.60    <1e-99
Sex: Boys                        0.412876    0.0047722   86.52    <1e-99
Test: Star_r & a1                0.215879    0.0117053   18.44    <1e-75
Test: S20_r & a1                -0.0689119   0.0110723   -6.22    <1e-09
```

```
Test: SLJ & a1                  -0.00711959  0.0103304    -0.69    0.4907
Test: BPT & a1                   0.306706    0.0118543    25.87    <1e-99
Test: Star_r & Sex: Boys        -0.260301    0.00666984  -39.03    <1e-99
Test: S20_r & Sex: Boys          0.0778728   0.00630514   12.35    <1e-34
Test: SLJ & Sex: Boys            0.0660908   0.0058801    11.24    <1e-28
Test: BPT & Sex: Boys            0.296268    0.00675837   43.84    <1e-99
a1 & Sex: Boys                   0.00194229  0.0138191     0.14    0.8882
Test: Star_r & a1 & Sex: Boys    0.0393094   0.0225936     1.74    0.0819
Test: S20_r & a1 & Sex: Boys    -0.0135135   0.0213581    -0.63    0.5269
Test: SLJ & a1 & Sex: Boys      -0.0196414   0.0199266    -0.99    0.3243
Test: BPT & a1 & Sex: Boys       0.0246167   0.0229        1.07    0.2824
```

6.2 Effects PCA w/ CPs

6.2.1 Random factor `Child`

```
m1_pca=MixedModels.PCA(m1, corr=true)
show(stdout, m1_pca.Child, stddevs=true)
```

```
Child =
Principal components based on correlation matrix
 (Intercept)    1.0      .      .      .      .
 Test: Star_r   0.11   1.0      .      .      .
 Test: S20_r    0.06  -0.51   1.0      .      .
 Test: SLJ      0.04   0.01  -0.36   1.0      .
 Test: BPT     -0.31   0.15  -0.2   -0.23   1.0

Normalized cumulative variances:
[0.3337, 0.6155, 0.8209, 0.9331, 1.0]

Component loadings
                PC1    PC2    PC3    PC4    PC5
 (Intercept)   -0.07   0.55  -0.61  -0.55  -0.07
 Test: Star_r   0.59   0.02  -0.48   0.42   0.49
 Test: S20_r   -0.7   -0.07  -0.11   0.04   0.7
 Test: SLJ      0.3    0.5    0.61  -0.27   0.46
 Test: BPT      0.27  -0.66  -0.05  -0.66   0.23
```

6.2.2 Random factor `School`

```
show(stdout, m1_pca.School, stddevs=true)
```

```
School =
Principal components based on correlation matrix
 (Intercept)    1.0      .      .      .      .      .      .
 Test: Star_r   0.14   1.0      .      .      .      .      .
 Test: S20_r   -0.14  -0.59   1.0      .      .      .      .
 Test: SLJ     -0.13   0.02  -0.5    1.0      .      .      .
```

```
 Test: BPT     -0.19  -0.04   0.06  -0.38   1.0     .      .
 a1             0.48  -0.03  -0.11  -0.01  -0.1    1.0     .
 Sex: Boys      0.09  -0.14   0.13   0.05  -0.19   0.25   1.0

Normalized cumulative variances:
[0.2756, 0.5077, 0.6969, 0.8091, 0.915, 0.975, 1.0]

Component loadings
                 PC1    PC2    PC3    PC4    PC5    PC6    PC7
 (Intercept)   -0.3    0.47   0.39  -0.35   0.01   0.63  -0.13
 Test: Star_r  -0.44  -0.29   0.39   0.14   0.53  -0.26  -0.45
 Test: S20_r    0.6    0.29  -0.12  -0.24   0.18  -0.13  -0.66
 Test: SLJ     -0.43  -0.17  -0.56   0.04  -0.39   0.19  -0.53
 Test: BPT      0.33  -0.22   0.47   0.55  -0.41   0.28  -0.25
 a1            -0.25   0.56   0.21   0.14  -0.45  -0.6   -0.08
 Sex: Boys     -0.04   0.47  -0.31   0.69   0.4    0.21   0.04
```

7 LMM with CPs for Test scores (Table 2)

7.1 LMM `m1L`

- The LMM `m1L` estimates CPs between test scores.
- This is a reparameterized version of the reference LMM `m1L`.

```
f1L = @formula zScore ~ 1 + Test*a1*Sex +
      (0+Test+a1+Sex | School) +  (0+Test | Child) + zerocorr(0+Test | Cohort);

m1L = fit(MixedModel, f1L, dat, contrasts=contr);
show(stdout, m1L)
```

```
Minimizing 6285  Time: 0 Time: 1:13:34 ( 0.70  s/it)
Linear mixed model fit by maximum likelihood
 zScore ~ 1 + Test + a1 + Sex + Test & a1 + Test & Sex + a1 & Sex + Test & a1 & Sex +
 (0 + Test + a1 + Sex | School) + (0 + Test | Child) + MixedModels.ZeroCorr((0 + Test | Cohort))
   logLik     -2 logLik       AIC         AICc          BIC
 -657029.5083 1314059.0165 1314197.0165 1314197.0349 1314967.8427

Variance components:
            Column     Variance  Std.Dev.   Corr.
Child    Test: Run     0.5482510 0.7404397
         Test: Star_r  0.5756597 0.7587224 +0.56
         Test: S20_r   0.5813383 0.7624554 +0.61 +0.67
         Test: SLJ     0.6043382 0.7773919 +0.59 +0.65 +0.77
         Test: BPT     0.5133581 0.7164902 +0.25 +0.45 +0.43 +0.50
School   Test: Run     0.1010168 0.3178314
         Test: Star_r  0.1403873 0.3746830 +0.35
         Test: S20_r   0.0957053 0.3093627 +0.34 +0.33
         Test: SLJ     0.0643472 0.2536676 +0.37 +0.39 +0.44
         Test: BPT     0.0576104 0.2400216 +0.19 +0.18 +0.28 +0.26
         a1            0.0093963 0.0969345 +0.44 +0.34 +0.28 +0.32 +0.21
         Sex: Boys     0.0024040 0.0490303 +0.12 -0.05 +0.11 +0.19 -0.04 +0.25
Cohort   Test: Run     0.0028538 0.0534213
```

```
        Test: Star_r  0.0013662 0.0369620   .
        Test: S20_r   0.0037516 0.0612506   .     .
        Test: SLJ     0.0012754 0.0357126   .     .     .
        Test: BPT     0.0006946 0.0263560   .     .     .     .
Residual              0.2966384 0.5446452
 Number of obs: 525126; levels of grouping factors: 108295, 515, 9

  Fixed-effects parameters:

                                   Coef.  Std. Error       z  Pr(>|z|)

(Intercept)                   -0.0383779  0.0115183    -3.33    0.0009
Test: Star_r                   0.0186882  0.0283087     0.66    0.5092
Test: S20_r                   -0.0333636  0.030085     -1.11    0.2674
Test: SLJ                      0.0328335  0.0274972     1.19    0.2325
Test: BPT                      0.00462939 0.0204412     0.23    0.8208
a1                             0.271406   0.0085921    31.59    <1e-99
Sex: Boys                      0.412876   0.00477165   86.53    <1e-99
Test: Star_r & a1              0.215399   0.0117123    18.39    <1e-74
Test: S20_r & a1              -0.06849    0.0110738    -6.18    <1e-09
Test: SLJ & a1                -0.00665803 0.0103309    -0.64    0.5193
Test: BPT & a1                 0.305842   0.0118581    25.79    <1e-99
Test: Star_r & Sex: Boys      -0.260285   0.00666989  -39.02    <1e-99
Test: S20_r & Sex: Boys        0.077859   0.00630521   12.35    <1e-34
Test: SLJ & Sex: Boys          0.0660713  0.00588016   11.24    <1e-28
Test: BPT & Sex: Boys          0.296291   0.00675835   43.84    <1e-99
a1 & Sex: Boys                 0.00194417 0.0138193     0.14    0.8881
Test: Star_r & a1 & Sex: Boys  0.0392733  0.0225938     1.74    0.0822
Test: S20_r & a1 & Sex: Boys  -0.0134742  0.0213583    -0.63    0.5281
Test: SLJ & a1 & Sex: Boys    -0.0195882  0.0199268    -0.98    0.3256
Test: BPT & a1 & Sex: Boys     0.0245743  0.0229       1.07    0.2832
```

7.2 Levels PCA w/ CPs

7.2.1 Random factor Child

```
m1L_pca=MixedModels.PCA(m1L, corr=true)
show(stdout, m1L_pca.Child, stddevs=true)
```

```
Child =
Principal components based on correlation matrix
 Test: Run     1.0    .     .     .    .
 Test: Star_r  0.56  1.0    .     .    .
 Test: S20_r   0.61  0.67  1.0    .    .
 Test: SLJ     0.59  0.65  0.77  1.0   .
 Test: BPT     0.25  0.45  0.43  0.5  1.0

Normalized cumulative variances:
[0.6472, 0.8017, 0.8809, 0.9559, 1.0]

Component loadings
```

```
                 PC1    PC2    PC3    PC4    PC5
 Test: Run     -0.42   0.53   0.7    0.22   0.01
 Test: Star_r  -0.47   0.02  -0.52   0.71  -0.07
 Test: S20_r   -0.49   0.13  -0.25  -0.44   0.69
 Test: SLJ     -0.5   -0.0   -0.13  -0.49  -0.71
 Test: BPT     -0.35  -0.84   0.39   0.09   0.11
```

7.2.2 Random factor School

```
show(stdout, m1L_pca.School, stddevs=true)
```

```
 School =
Principal components based on correlation matrix
 Test: Run      1.0     .      .      .      .      .      .
 Test: Star_r   0.35   1.0     .      .      .      .      .
 Test: S20_r    0.34   0.33   1.0     .      .      .      .
 Test: SLJ      0.37   0.39   0.44   1.0     .      .      .
 Test: BPT      0.19   0.18   0.28   0.26   1.0     .      .
 a1             0.44   0.34   0.28   0.32   0.21   1.0     .
 Sex: Boys      0.12  -0.05   0.11   0.19  -0.04   0.25   1.0

Normalized cumulative variances:
[0.3772, 0.5359, 0.6587, 0.7665, 0.8548, 0.933, 1.0]

Component loadings
                 PC1    PC2    PC3    PC4    PC5    PC6    PC7
 Test: Run     -0.43   0.06  -0.31   0.26  -0.6    0.43  -0.32
 Test: Star_r  -0.4   -0.28  -0.47  -0.13   0.55  -0.18  -0.44
 Test: S20_r   -0.42  -0.11   0.24  -0.49  -0.45  -0.56   0.01
 Test: SLJ     -0.45   0.02   0.11  -0.43   0.24   0.57   0.47
 Test: BPT     -0.29  -0.39   0.71   0.43   0.15   0.08  -0.21
 a1            -0.42   0.26  -0.18   0.54   0.11  -0.37   0.53
 Sex: Boys     -0.16   0.83   0.29  -0.09   0.2   -0.03  -0.40
```

8 LMM with quadratic trends of age

Adding a quadratic trend for age to the reference LMM `m1` did not significantly increase the goodness of fit.

```
f1_agesq = @formula zScore ~ 1 + Test*(a1+a2)*Sex  +
           (1+Test+a1+Sex | School) +  (1+Test | Child) + zerocorr(1+Test | Cohort);

m1_agesq = fit(MixedModel, f1_agesq, dat, contrasts=contr);
show(stdout, m0_agesq);

show(stdout, MixedModels.likelihoodratiotest(m1, m1_agesq))
```

```
Model Formulae
1: zScore ~ 1 + Test + a1 + Sex +
            Test & a1 + Test & Sex + a1 & Sex +
            Test & a1 & Sex +
```

```
           (1 + Test + a1 + Sex | School) + (1 + Test | Child) +
            MixedModels.ZeroCorr((1 + Test | Cohort))
2: zScore ~ 1 + Test + a1 + a2 + Sex +
            Test & a1 + Test & a2 + Test & Sex + a1 & Sex + a2 & Sex +
            Test & a1 & Sex + Test & a2 & Sex +
           (1 + Test + a1 + Sex | School) + (1 + Test | Child) +
            MixedModels.ZeroCorr((1 + Test | Cohort))

     model-dof     -2 logLik       ²    ²-dof  P(> ²)

[1]         69  1314070.9604
[2]         79  1314061.7724  9.1879      10  0.5144
```

9 LMM with Age x Sex interactions nested in levels of Test

- The nested LMM `m1LL` estimates the age x sex interaction for each (level of) test.
- This is a reparameterized version of the reference LMM `m1`.

The following details about this LMM are not reported in the paper, only here.

```
f1LL_nested = @formula zScore ~ 0 + Test & (a1*Sex) +
            (0+Test+a1+Sex | School) +  (0+Test | Child) + zerocorr(0+Test | Cohort);

m1LL_nested = fit(MixedModel, f1LL_nested, dat, contrasts=contr);
show(stdout, m1LL_nested)
```

```
Linear mixed model fit by maximum likelihood
 zScore ~ 0 + Test & a1 + Test & Sex + Test & a1 & Sex +
         (0 + Test + a1 + Sex | School) + (0 + Test | Child) +
          MixedModels.ZeroCorr((0 + Test | Cohort))

    logLik     -2 logLik       AIC         AICc          BIC
 -657029.4875 1314058.9750 1314196.9750 1314196.9934 1314967.8011

Variance components:
            Column     Variance  Std.Dev.   Corr.
Child    Test: Run     0.5396732 0.7346246
         Test: Star_r  0.5671056 0.7530641 +0.56
         Test: S20_r   0.5727715 0.7568167 +0.62 +0.68
         Test: SLJ     0.5957455 0.7718455 +0.60 +0.66 +0.79
         Test: BPT     0.5047653 0.7104684 +0.25 +0.46 +0.44 +0.51
School   Test: Run     0.1005767 0.3171383
         Test: Star_r  0.1416812 0.3764057 +0.35
         Test: S20_r   0.0953173 0.3087351 +0.34 +0.33
         Test: SLJ     0.0645560 0.2540787 +0.37 +0.40 +0.44
         Test: BPT     0.0576309 0.2400644 +0.19 +0.19 +0.28 +0.26
         a1            0.0093726 0.0968123 +0.45 +0.34 +0.28 +0.32 +0.21
         Sex: Boys     0.0024128 0.0491199 +0.12 -0.05 +0.11 +0.19 -0.04 +0.25
Cohort   Test: Run     0.0029534 0.0543452
         Test: Star_r  0.0013685 0.0369930   .
         Test: S20_r   0.0037631 0.0613443   .     .
```

```
        Test: SLJ     0.0013055 0.0361312   .     .     .
        Test: BPT     0.0006859 0.0261901   .     .     .     .
Residual              0.3052439 0.5524888
 Number of obs: 525126; levels of grouping factors: 108295, 515, 9

  Fixed-effects parameters:
```

	Coef.	Std. Error	z	Pr(>|z|)
Test: Run & a1	0.0816709	0.0112849	7.24	<1e-12
Test: Star_r & a1	0.297056	0.0114161	26.02	<1e-99
Test: S20_r & a1	0.228565	0.0114096	20.03	<1e-88
Test: SLJ & a1	0.221929	0.0114894	19.32	<1e-82
Test: BPT & a1	0.527747	0.0110008	47.97	<1e-99
Test: Run & Sex: Girls	-0.291666	0.0234262	-12.45	<1e-34
Test: Star_r & Sex: Girls	-0.142937	0.0214637	-6.66	<1e-10
Test: S20_r & Sex: Girls	-0.215145	0.025084	-8.58	<1e-17
Test: SLJ & Sex: Girls	-0.215364	0.0171389	-12.57	<1e-35
Test: BPT & Sex: Girls	-0.3589	0.0146471	-24.50	<1e-99
Test: Run & Sex: Boys	0.197035	0.0236004	8.35	<1e-16
Test: Star_r & Sex: Boys	0.0854815	0.0213975	3.99	<1e-04
Test: S20_r & Sex: Boys	0.0911323	0.0252317	3.61	0.0003
Test: SLJ & Sex: Boys	0.156983	0.0174472	9.00	<1e-18
Test: BPT & Sex: Boys	0.309738	0.0146131	21.20	<1e-98
Test: Run & a1 & Sex: Boys	-0.0184637	0.0197425	-0.94	0.3497
Test: Star_r & a1 & Sex: Boys	0.0208101	0.0200437	1.04	0.2992
Test: S20_r & a1 & Sex: Boys	0.00733641	0.0200146	0.37	0.7140
Test: SLJ & a1 & Sex: Boys	-0.012253	0.020209	-0.61	0.5443
Test: BPT & a1 & Sex: Boys	0.0123209	0.0191743	0.64	0.5205

None of the five interaction terms (last five lines in table above) are significant.

10 A few standard observation-level residual plots

The figure is based on a random sample of 50,000 residuals (i.e., ~ 10%); it takes too long otherwise. Repeated execution did not reveal any disturbing patterns.

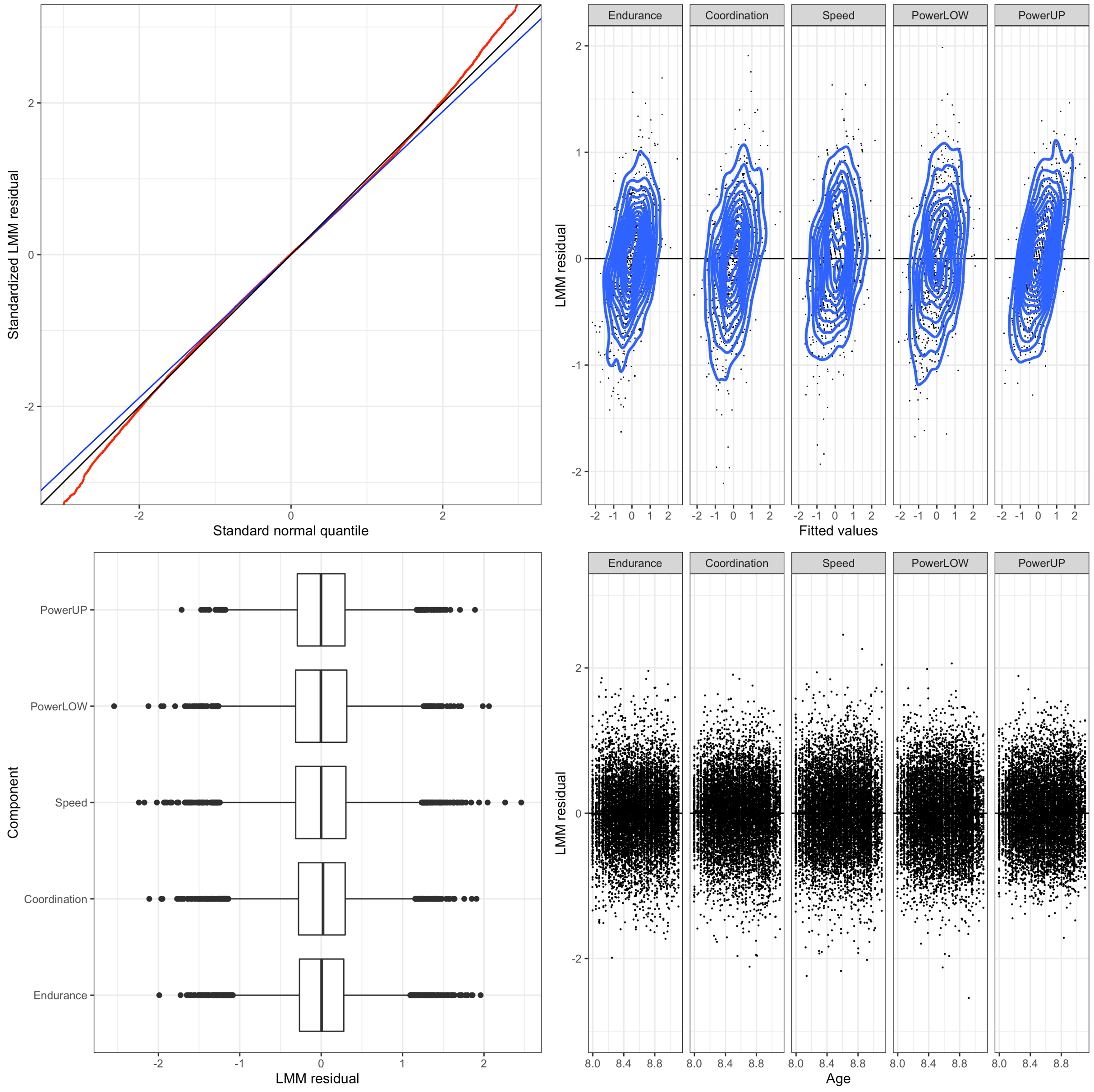

11 Julia and package versions

```
using Pkg
show(stdout, Pkg.status())
using InteractiveUtils
show(stdout, versioninfo(verbose=true))
```

```
Status `~/Google Drive/git_projects/SMLP2020/Project.toml`
  [a93c6f00] DataFrames v1.2.0
  [75880514] DataFrameMacros v0.1.0
  [ff71e718] MixedModels v4.0.0
  [6f49c342] RCall v0.13.12
  [2913bbd2] StatsBase v0.33.8

Julia Version 1.6.2
Commit 1b93d53fc4 (2021-07-14 15:36 UTC)
Platform Info:  OS: macOS (x86_64-apple-darwin18.7.0)
```

12 References

- Bates, D., Alday, P., Kleinschmidt, D., et al. (2021, July 16). JuliaStats/MixedModels.jl: v4.0.0. Zenodo. https://zenodo.org/record/5111017 doi: 10.5281/zenodo.5111017
- Bates, D., Kliegl, R., Vasishth, S., & Baayen, H. (2015). Parsimonious mixed models. arXiv:1506.04967 [stat.ME].
- Bates,D., Maechler, M., Bolker, B., Walker, S. (2015). Fitting Linear Mixed-Effects Models Using lme4. *Journal of Statistical Software*, *67*, 1-48. doi:10.18637/jss.v067.i01. (Version 1.1.26)
- Bolker, B., & Robinson, R. (2020). broom.mixed: Tidying Methods for Mixed Models. R package version 0.2.6. https://CRAN.R-project.org/package=broom.mixed

- Bezanson, J., Edelman, A., Karpinski, S., Shah, B. V. (2017). Julia: A fresh approach to numerical computing. *SIAM review*, *59*, 65-98. <URL: https://doi.org/10.1137/141000671>
- R Core Team (2021). R: A language and environment for statistical computing. R Foundation for Statistical Computing, Vienna, Austria. URL https://www.R-project.org/. (Version 4.04)
- Wickham et al., (2019). Welcome to the tidyverse. Journal of Open Source Software, 4(43), 1686, https://doi.org/10.21105/joss.01686 (Version 1.3.0)
- Wilke, C.O. (2020). cowplot: Streamlined Plot Theme and Plot Annotations for 'ggplot2'. R package version 1.1.1. https://CRAN.R-project.org/package=cowplot (Version 1.1.1)

Supplement B: Reanalyses of Golle et al. (2015) for manuscript: 'Age and sex effects in physical fitness components of 108,295 third graders including 515 primary schools and 9 cohorts' written by Fühner, T., Granacher, U., Golle, K., & Kliegl, R.

2021-02-16 (updated: 2021-07-28 10:03:40)

Contents

1 Background **1**

1.1 Reference . . . 1

1.2 Overview . . . 2

2 Longitudinal and cross-sectional ontogenesis (2006 to 2009) **2**

3 Setup **2**

4 Figure 1 **3**

5 Longitudinal LMM: age (2nd-order) x Sex within Test **3**

5.1 LMM . . . 3

5.2 Summary . . . 4

6 Cross-sectional LMMs **4**

6.1 LMM for 9 to 10 year old children (insets of Figure 1) . . . 4

7 References and versions **6**

1 Background

1.1 Reference

This script is for a Supplement that accompanies Fühner, T., Granacher, U., Golle, K., & Kliegl, R. (2021). *Age and sex effects in physical fitness components of 108,295 third graders including 515 primary schools and 9 cohorts.* Scripts and data are available in OSF repository: https://osf.io/2d8rj/

1.2 Overview

- Figure 1 of paper (only in script)
- Reanalysis of longitudinal profiles
- Analysis of cross-sectional age effect and age x sex interactions
  - aggregating across tests within the four years of assessment
  - separately for each test within each of the four years of assessment

2 Longitudinal and cross-sectional ontogenesis (2006 to 2009)

We reanalyzed the longitudinal data from the first EMOTIKON project reported in Golle et al. (2015). Physical Fitness Percentiles of German Children Aged 9–12 Years: Findings from a Longitudinal Study. *PLoS ONE, 10,* 11 (doi:10.1371/journal.pone.0142393). The primary purpose was to use these data for Figure 1 in the paper.

We test age, sex, and age x sex effects for the longitudinal data (i.e., across four assessments). This is basically a reanalysis of the paper with LMMs.

The longitudinal sample comprised 152 boys and 88 girls. Therefore, we examined age x sex interactions for five fitness components, corresponding to the ones used in the paper **within** the four years of assessment, that is look at the data also from this cross-sectional perspective. Note that the specific tests used in this first Emotikon study were replaced with different tests for the same components of physical fitness in the current one.

In the **longitudinal LMM**, there was significant growth for each test (decelerating for `Endurance` and `Coordination`) Boys outperformed girls; this sex effect was not significant for `Coordination`. The interaction between age and sex was significant only for `PowerUP` (i.e., `BPT`) with larger growth for boys than girls.

With four **cross-sectional LMMs** (i.e., within each year of assessment), we also tested the between-subject age differences. There were no age effects or age x sex interactions – neither when aggregating across the five tests nor within any of the five tests. And this was true for all four years of assesseement.

3 Setup

Variables for this re-analysis were extracted from a comprehensive data file; tests were z-transformed with mean and standard deviation of first assessment in 2006.

The following variables are used.

1. School: 27 levels
2. Child: 240 levels; all children were tested four times (2006 - 2009)
3. Sex: "Boys" (n=152), "Girls" (n=88)
4. age: test date - middle of month of birthdate
5. year: 2006 - 2009
6. Test: 5 levels:
   - Endurance (`Run`, cardiorespiratory endurance): 6 min run test [m]; to nearest 9m in 9x18m field
   - Coordination (`Star_r`): star run test [m/s]; 9x9m field, 4 x diagonal = 50.912 m
   - Speed(`S50_r`): 50-m linear sprint test [m/s]
   - PowerLOW (`THJ`; Power of lower limbs): triple hop jump test [cm]
   - PowerUP (`BPT`; Power of upper limbs): 1-kg ball push test [m] with one arm
7. zScore

4 Figure 1

This code chunk generates Figure 1 in the paper.

5 Longitudinal LMM: age (2nd-order) x Sex within Test

5.1 LMM

5.1.1 Parsimonious LMM selection

Parsiomonious LMM section followed the general recommendations of Bates et al. (2015). Details are available in script on osf repo.

5.1.2 Parsimoious longitudinal LMM for 9.00 to 12.99 year old

```
lmm_long_simple <- lmer(zScore ~ 1 + Test/(Sex*a1 + a2) +
                               (1 + a1 | Child) + (1 + a1 + a2 || School),
                        data=dat, REML=FALSE, control=lmerControl(calc.derivs=FALSE))
print(summary(lmm_long_simple), cor=FALSE)
```

```
## Linear mixed model fit by maximum likelihood  ['lmerMod']
## Formula: zScore ~ 1 + Test/(Sex * a1 + a2) + (1 + a1 | Child) + ((1 |
##     School) + (0 + a1 | School) + (0 + a2 | School))
##    Data: dat
## Control: lmerControl(calc.derivs = FALSE)
##
##      AIC      BIC   logLik deviance df.resid
##  11376.7  11583.9  -5656.3  11312.7     4768
##
## Scaled residuals:
##     Min      1Q  Median      3Q     Max
## -4.4644 -0.6102 -0.0037  0.5852  4.9275
##
## Random effects:
##  Groups   Name        Variance Std.Dev. Corr
##  Child    (Intercept) 0.518560 0.72011
##           a1          0.008501 0.09220  0.56
##  School   (Intercept) 0.030732 0.17531
##  School.1 a1          0.004415 0.06645
##  School.2 a2          0.004464 0.06681
##  Residual             0.517498 0.71937
## Number of obs: 4800, groups:  Child, 240; School, 27
##
## Fixed effects:
##                                  Estimate Std. Error t value
## (Intercept)                      0.884625   0.062139  14.236
## Test2-1                          0.590932   0.051044  11.577
## Test3-2                         -0.358435   0.051044  -7.022
## Test4-3                          0.272625   0.051044   5.341
## Test5-4                          0.167039   0.051044   3.272
```

```
## TestEndurance:Sex2-1           0.636114   0.109353   5.817
## TestCoordination:Sex2-1        0.269003   0.109353   2.460
## TestSpeed:Sex2-1               0.232640   0.109353   2.127
## TestMuscle power low:Sex2-1    0.277652   0.109353   2.539
## TestMuscle power up:Sex2-1     1.018715   0.109353   9.316
## TestEndurance:a1               0.201382   0.026252   7.671
## TestCoordination:a1            0.549028   0.026252  20.914
## TestSpeed:a1                   0.434773   0.026252  16.561
## TestMuscle power low:a1        0.569085   0.026252  21.678
## TestMuscle power up:a1         0.778939   0.026252  29.671
## TestEndurance:a2              -0.087447   0.024584  -3.557
## TestCoordination:a2           -0.080338   0.024584  -3.268
## TestSpeed:a2                  -0.027774   0.024584  -1.130
## TestMuscle power low:a2       -0.040876   0.024584  -1.663
## TestMuscle power up:a2        -0.018337   0.024584  -0.746
## TestEndurance:Sex2-1:a1       -0.060012   0.044328  -1.354
## TestCoordination:Sex2-1:a1    -0.003030   0.044328  -0.068
## TestSpeed:Sex2-1:a1           -0.064772   0.044328  -1.461
## TestMuscle power low:Sex2-1:a1 -0.003465   0.044328  -0.078
## TestMuscle power up:Sex2-1:a1   0.099951   0.044328   2.255
```

5.2 Summary

- Note
  - "linear growth" refers to significant linear trend of age
  - "decelerating growth" refers to significant negative quadratic trend of age.
- Age and Sex: Boys > girls and significant linear growth across age for all tests. In addition, decelerating growth for Endurance and Coordination, and a significantly larger linear growth for boys than girls for `PowerUP`
- Test contrasts
  - Coordination grows more than Endurance and more than Speed
  - PowerLOW grows more than Speed, but less than PowerUP
- Variance components and correlation parameters
  - Child: There are reliable individual differences between children's GM and their linear rate of growth and they correlate positively (.56; i.e., Matthew effect.)
  - School: There are reliable differences between schools's GM and between their second-order growth-rates; no support for correlation parameters.

6 Cross-sectional LMMs

6.1 LMM for 9 to 10 year old children (insets of Figure 1)

6.1.1 Parsimonious LMM selection

Parsiomonious LMM section followed the general recommendations of Bates et al. (2015). Details are available in script on osf repo.

```
contrasts(dat06$Sex) <- (-1)*MASS::contr.sdif(2)
dat06$sex <- ifelse(dat06$Sex == "Boys", +1/2, -1/2)
contrasts(dat06$Test) <- MASS::contr.sdif(5)
dat06$a1 <- dat06$age - 9.5  # center
dat06$a2 <- dat06$a1^2

lmm_cross06 <- lmer(zScore ~ 1 + Test/((a1+a2)*Sex) + (1  | Child) + (1 | School),
                         data=dat06, REML=FALSE, control=lmerControl(calc.derivs=FALSE))
summary(rePCA(lmm_cross06))
print(summary(lmm_cross06))

# ... further simplification: only main effects of age and sex within Test as fixed effects
lmm_cross_simple06 <- lmer(zScore ~ 1 + Test/(a1+Sex) + (1  | Child) + (1 | School),
                         data=dat06, REML=FALSE, control=lmerControl(calc.derivs=FALSE))

anova(lmm_cross_simple06, lmm_cross06)

print(summary(lmm_cross_simple06), cor=FALSE)
```

6.1.2 Parsimonious crosssectional LMMs

We test the age effect and age x sex interaction nested within each of the four assessments. Note insets in Figure 1 show only data from first assessment.

```
dat$Year <- factor(dat$year)

lmm_cross_simple1 <- lmer(zScore ~ 1 + Year/(Test*a1*Sex) + (1  | Child) + (1 | School),
                         data=dat, REML=FALSE, control=lmerControl(calc.derivs=FALSE))

# without age effect
lmm_cross_noage1 <- lmer(zScore ~ 1 + Year/(Test*Sex) + (1  | Child) + (1 | School),
                         data=dat, REML=FALSE, control=lmerControl(calc.derivs=FALSE))

anova(lmm_cross_noage1, lmm_cross_simple1)
```

6.1.2.1 Age and sex nested within years

```
## Data: dat
## Models:
## lmm_cross_noage1: zScore ~ 1 + Year/(Test * Sex) + (1 | Child) + (1 | School)
## lmm_cross_simple1: zScore ~ 1 + Year/(Test * a1 * Sex) + (1 | Child) + (1 | School)
##                    npar   AIC   BIC  logLik deviance  Chisq Df Pr(>Chisq)
## lmm_cross_noage1    43 11480 11758 -5696.9    11394
## lmm_cross_simple1   83 11510 12048 -5672.2    11344 49.382 40     0.1469
```

Summary

Removing age from the LMM does not significantly reduce the goodness of fit. There is no evidence for significant age effect and also no evidence for significant age x sex interactions when aggregating across the five tests within any of the four assessments.

```r
lmm_cross_simple2 <- lmer(zScore ~ 1 + Year/Test/(a1*Sex) + (1  | Child) + (1 | School),
                            data=dat, REML=FALSE, control=lmerControl(calc.derivs=FALSE))

# without age effect
lmm_cross_noage2 <- lmer(zScore ~ 1 + Year/Test/Sex + (1  | Child) + (1 | School),
                            data=dat, REML=FALSE, control=lmerControl(calc.derivs=FALSE))

anova(lmm_cross_noage2, lmm_cross_simple2)
```

6.1.2.2 Age and sex nested within tests nested within years

```
## Data: dat
## Models:
## lmm_cross_noage2: zScore ~ 1 + Year/Test/Sex + (1 | Child) + (1 | School)
## lmm_cross_simple2: zScore ~ 1 + Year/Test/(a1 * Sex) + (1 | Child) + (1 | School)
##                   npar   AIC   BIC  logLik deviance  Chisq Df Pr(>Chisq)
## lmm_cross_noage2    43 11480 11758 -5696.9    11394
## lmm_cross_simple2   83 11510 12048 -5672.2    11344 49.382 40     0.1469
```

Summary

Removing age from the LMM does not significantly reduce the goodness of fit. Thus, there is no evidence for significant age effects and also no evidence for significant age x sex interactions when tested separately for each of the five tests within any of the four assessments.

7 References and versions

- Bates, D., Kliegl, R., Vasishth, S., & Baayen, H. (2015). Parsimonious mixed models. arXiv:1506.04967 [stat.ME].
- Bates, D., Maechler, M., Bolker, B., & Walker, S. (2015). Fitting Linear Mixed-Effects Models Using lme4. Journal of Statistical Software, 67(1), 1-48. doi:10.18637/jss.v067.i01. (Version 1.1.26)
- R Core Team (2021). R: A language and environment for statistical computing. R Foundation for Statistical Computing, Vienna, Austria. URL https://www.R-project.org/. (Version 4.04)
- Wickham H. et al., (2019). Welcome to the tidyverse. Journal of Open Source Software, 4(43), 1686, https://doi.org/10.21105/joss.01686 (Version 1.3.0)
- Wilke, C.O. (2020). cowplot: Streamlined Plot Theme and Plot Annotations for 'ggplot2'. R package version 1.1.1. https://CRAN.R-project.org/package=cowplot (Version 1.1.1)
